# Rnf165/Ark2C Enhances BMP-Smad Signaling to Mediate Motor Axon Extension

**DOI:** 10.1371/journal.pbio.1001538

**Published:** 2013-04-16

**Authors:** Claire E. Kelly, Efstathia Thymiakou, James E. Dixon, Shinya Tanaka, Jonathan Godwin, Vasso Episkopou

**Affiliations:** Division of Brain Sciences, Faculty of Medicine, Imperial College London, London, United Kingdom; University of Basel, Switzerland

## Abstract

Little is known about extrinsic signals required for the advancement of motor neuron (MN) axons, which extend over long distances in the periphery to form precise connections with target muscles. Here we present that Rnf165 (Arkadia-like; Arkadia2; Ark2C) is expressed specifically in the nervous system and that its loss in mice causes motor innervation defects that originate during development and lead to wasting and death before weaning. The defects range from severe reduction of motor axon extension as observed in the dorsal forelimb to shortening of presynaptic branches of the phrenic nerve, as observed in the diaphragm. Molecular functional analysis showed that in the context of the spinal cord Ark2C enhances transcriptional responses of the Smad1/5/8 effectors, which are activated (phosphorylated) downstream of Bone Morphogenetic Protein (BMP) signals. Consistent with Ark2C-modulated BMP signaling influencing motor axons, motor pools in the spinal cord were found to harbor phosphorylated Smad1/5/8 (pSmad) and treatment of primary MN with BMP inhibitor diminished axon length. In addition, genetic reduction of BMP-Smad signaling in *Ark2C* ^+/−^ mice caused the emergence of *Ark2C* ^−/−^-like dorsal forelimb innervation deficits confirming that enhancement of BMP-Smad responses by Ark2C mediates efficient innervation. Together the above data reveal an involvement of BMP-Smad signaling in motor axon advancement.

## Abstract 2

Efficient extension of motor axons into the limb during development requires enhancement of BMP signaling responses by the ubiquitin ligase Ark2C.

## Author Summary

Motor neurons control movement via long axons that extend from the spinal cord to muscles as far as in distant limbs. Little is known about factors that regulate this extensive axonal growth in the periphery. Here we report that the ubiquitin ligase Ark2C (Arkadia2) is expressed in neurons and can serve to amplify neuronal responses to specific signals. We find that these signals belong to the Bone Morphogenetic Protein (BMP) family of secreted factors, which are highly expressed in the periphery and known to regulate the development of the limbs. Loss of Ark2C gene function in mice results in inefficient growth of motor axons to distant muscles, and we show that this process is regulated by BMP signaling. Ark2C targets BMP inhibitors for destruction, and therefore the presence of Ark2C helps to enhance BMP signaling, which in turn is necessary for the innervation of distal muscles. Our experiments reveal a previously unknown function of BMP in motor axon growth and describe a molecular mechanism for how axons and limbs coordinate their growth.

## Introduction

The assembly of neural circuits is complex and highly specific. Progenitors and early postmitotic neurons acquire an intrinsic genetic program that controls circuit assembly steps including axonal path finding and synaptic partner recognition [1]–[3]. In the brain, extrinsic signals that control axon initiation and advancement are beginning to be identified [4],[5]. Motor axons follow precise paths through peripheral tissues [6], and extrinsic signals acting directly upon the motor axons [7] or upon adjacent sensory axons [8] have been implicated in steering their advancement. Additionally, intrinsic properties of different MN subtypes produce varying responses to extrinsic signals creating a highly specific pattern of innervation [9]. However, the developing peripheral tissue expresses many cytokines for its own development and the role of many of these in the manipulation of motor axon growth has not been fully addressed.

Transforming Growth Factor (TGF) β signaling is essential for embryonic development and adult tissue homeostasis in both vertebrates and invertebrates. BMP and Nodal/Activin are distinct classes of ligands within the TGFβ cytokine family; they signal through serine/threonine kinase receptor complexes, composed of type I and type II receptors, which activate by phosphorylation the Smad1/5/8 (pSmad1/5/8) and Smad2/3 (pSmad2/3) effectors, respectively. PSmads complex with Smad4 to translocate to the nucleus where they function as transcription factors [10]. Alternatively, TGFβ ligands can signal in a Smad-independent fashion [11]. Loss of function mutations of most components of the signaling pathway in mice result in multiple defects and early embryonic lethality [12], preventing the elucidation of their role in later aspects of development such as neuronal connectivity. Nevertheless emerging evidence supports a role, particularly for the BMP class of ligands, in axon guidance [13]. Smad-independent BMP signaling is associated with growth cone collapse and axon repulsion in the spinal cord [14],[15]. Less clear is the role of BMP-Smad signaling in connectivity, particularly of axons extending in the periphery. BMP/Smad1 signaling has been shown to have a role in sensory neuron axon regeneration in adult mice and axon regrowth in cultured dorsal root ganglia (DRG) [16],[17], but its role in neuromuscular connectivity during development has not been addressed. Moreover, ligands released by peripheral synaptic targets have also been shown to activate neuronal Smads in a retrograde manner and specify cellular identity within sympathetic and trigeminal sensory neurons [18],[19]. Retrograde Smad-dependent BMP signaling has also been shown to be required for MN synapse growth and plasticity in *Drosophila* [20]–[22]. However, the role of this pathway in mammalian MNs remains unknown.

Nodal/Activin and BMP ligands are well-known for their dose-dependent effects during development and for the multiple mechanisms, both extracellular and intracellular, that regulate their signaling activity [23]–[30]. Key intracellular negative regulators include the inhibitory Smad6/7 and the nuclear co-repressors Ski and SnoN. Smad6/7 mediate the degradation of receptors and reduce the phosphorylation of effector Smads, while Ski/SnoN directly interact with the pSmad/Smad4 complex and recruit histone deacetylases to the promoters of target genes, thereby inhibiting transcription [29]. As these negative regulators are upregulated by Smads, the pathway activates a negative feedback loop “auto shut-off” mechanism. The degradation of such intracellular repressors provides a mechanism to reduce negative feedback and the requirement for continuous stimulation by the ligand. Arkadia/RNF111, an E3 ubiquitin ligase, mediates the ubiquitin-proteasome degradation of Smad6/7 and Ski/SnoN [31]–[33]. Arkadia is required for head/anterior development in the mammalian embryo [34],[35], which also depends on the establishment of high Nodal-Smad2/3 signaling [30]. Interestingly, Arkadia specifically acts to derepress the Smad2/3 effectors because it removes Ski/SnoN only when they block these effectors. However, a similar mechanism for derepression of Smad1/5/8 and consequent intracellular enhancement of their transcriptional responses remained unknown.

Here we present the identification and molecular characterization of RNF165/Ark2C, an E3 ubiquitin ligase with homology to Arkadia that derepresses specifically Smad1/5/8 responses. *Ark2C* is expressed in the nervous system in mice, and its loss leads to MN axon extension and muscle innervation deficits. In *Ark2C* ^+/−^ mice genetic reduction of BMP signaling by removal of one allele of BMPRII causes dorsal forelimb innervation defects such as those observed in *Ark2C* ^−/−^ mice. A similar phenotype is observed in *Ark2C* ^+/−^ mice upon deletion of Smad8, an effector with a limited region of expression in the nervous system, which in the spinal cord includes subsets of MN. These results along with findings that limb motor pools in the spinal cord harbor nuclear phosphorylated Smad1/5/8, and that treatment of MN in culture with BMP and its inhibitors enhances and reduces axon length respectively, support a role for this pathway in axon elongation. Furthermore, it appears that in a subset of MN this role of BMP depends on intracellular enhancement of the pathway by Ark2C.

## Results

### A Gene-Trap Insertion Disrupts Ark2C and Reveals Its Expression in the Nervous System

**Figure 1:**
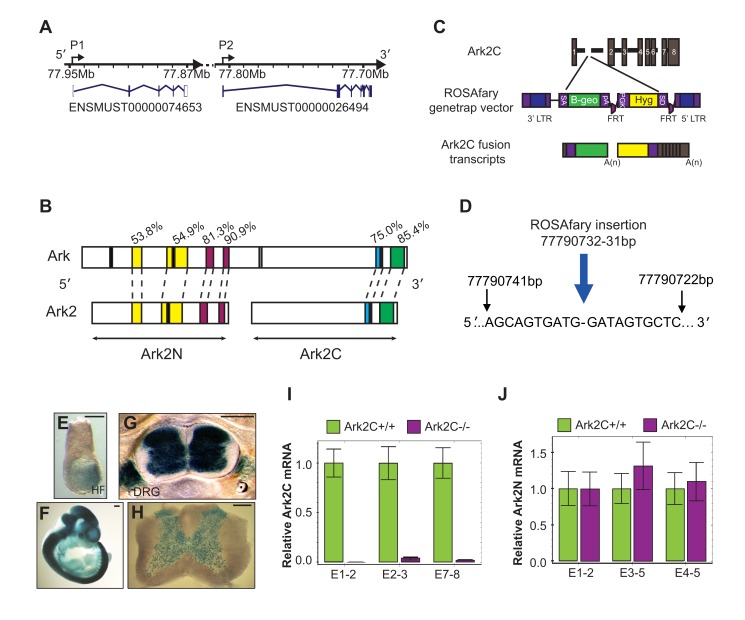
A novel Arkadia-like gene, Ark2C, is specifically expressed in the nervous system. (A) Schematic representation of the Ark2 locus (Chromosome 18:E3) showing two promoters (P1 and P2) and the transcripts corresponding to Ark2N and C (Ensembl release 63, June 2011). (B) Homologous domains of Arkadia and Ark2: serine rich domains (yellow), nuclear localization signals (black), Arkadia homology domains (red), NRG-TIER domain (blue), and a RING domain (green). Conservation as indicated by the percentages. (C) Schematic representation of ROSAfary gene-trap insertion into the first intron of Ark2C and the expected resulting fusion transcripts. (D) Sequence flanking the insertion site of the ROSAfary gene-trap in intron1 of Ark2C. Intron1 is 80,577 bp long, and the gene-trap insertion is 13,048 bp from exon1. (E–H) X-gal staining showing *Ark2C* expression (E) at embryonic day 7.5 (E7.5), (F) at E11.5, (G) at E16.5, and (H) in the adult. (G) and (H) are vibrotome transverse sections. HF, Headfold; DRG, dorsal root ganglia; scale bars = 100 µm (E) and 500 µm (F–H). (I) *Ark2C* expression in the embryonic brain of mice wild type (*wt*) and homozygous for the P9-3f gene-trap measured by quantitative RT-PCR. Transcripts from exon (E) 1–2 span the gene-trap insertion site and from exon7–8 include the NRG-TIER and RING domains. *N* = 3 embryos of each genotype. Error bars represent ±SD. (J) *Ark2N* expression in mice *wt* and homozygous P9-3f gene-trap measured by quantitative RT-PCR. Error bars represent ±SD. The gene downstream of *Ark2C* is *Loxhd1*. Mutations in this gene [74] do not exhibit similar to the P9-3f phenotypes (see Figures 2–4), indicating that the P93F gene trap is Ark2C gene specific.

The Arkadia-like locus contains two tandem promoters, separated by poly-adenylation sites, expressing two different genes (Figure 1A): here named Arkadia2N (Ark2N), with homology to the N-terminus of Arkadia, and Arkadia2C (Ark2C), with homology to the C-terminus of Arkadia. Ark2C contains a RING domain previously annotated as Rnf165, which is 85% identical to that of Arkadia. Other highly conserved domains include a nuclear localization signal and the NRG-TIER domain, which is involved in substrate interactions (Figure 1B) [32],[33],[35]. Sequence-alignment detected two additional Arkadia-like genes (Figure S1A); both were found to contain stop codons and were therefore considered to be pseudogenes.

To address the function of Ark2C in vivo we generated mice carrying a gene-trap in the first intron (Figure 1C and D). The expression of the gene-trap *lacZ*-reporter, which depends on the endogenous Ark2C promoter, revealed that Ark2C is expressed specifically in the nervous system (Figure 1E and F and Figure S1B), including the spinal cord in both embryo (Figure 1G) and adult (Figure 1H). Quantitative RT-PCR analysis of wild-type (*wt*) and homozygous (*Ark2C* ^−/−^) embryonic brain RNA showed loss of expression in homozygous mutants, indicating that the gene-trap disrupts the transcription of *Ark2C* throughout the gene (Figure 1I) producing a null mutation. On the contrary, the gene trap does not affect the expression of the adjacent *Ark2N* as shown by quantitative RT-PCR (Figure 1J). Therefore, this gene-trap strain can be used for Ark2C-specific loss of function studies.

### Ark2C Is Involved in MN Neuromuscular Connectivity

**Figure 2:**
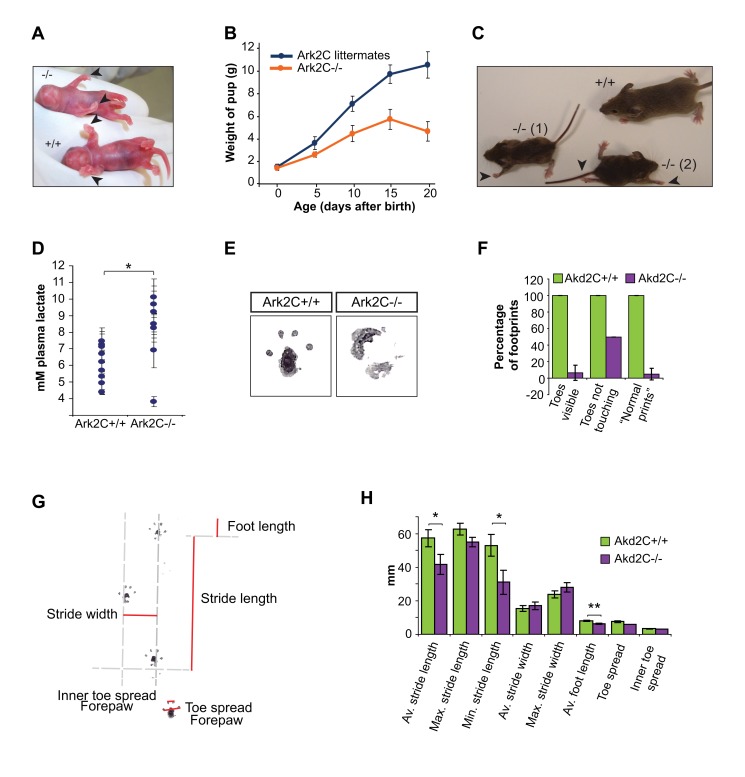
*Ark2C* ^−/−^ mice exhibit abnormal forelimb posture and movement, inefficient breathing and feeding. (A) *wt* (+/+) and *Ark2C* ^−/−^ (−/−) mice at postnatal day zero (P0). Black arrowheads indicate the relaxed paw posture. (B) Growth curves of *Ark2C* ^−/−^ mice and their littermates between P0 and P20. Error bars represent ±SD between individuals. (C) P21 *Ark2C* ^−/−^ mice and littermate, showing (1) less and (2) more severe phenotypes. Black arrowheads, defective limbs. (D) Blood plasma lactate concentrations from 9 *wt* and 7 *Ark2C* ^−/−^ mice at P21 and adult. Error bars represent ±SD of three readings for each individual; * *p*<0.05. (E) Representative footprints from 4 *Ark2C* ^+/+^ and 2 *Ark2C* ^−/−^ adult mice. (F) Histograms showing the percentage of footprints according to how clear the toe prints are. (G) Pawprint trail showing measurements analyzed. (H) Histograms showing results of pawprint analysis from 9 *wt* and 7 *Ark2C* ^−/−^ mice. Error bars represent ±SD between individuals; * *p*<0.05; ** *p*<0.01.

Approximately 10% of *Ark2C* ^−/−^ mice die at birth. The remaining *Ark2C* ^−/−^ pups are the same size as their littermates at birth (Figure 2A), however they fail to thrive and grow, reaching only 50% of the size of their siblings at postnatal day 15 (Figure 2B and C). Null pups are always born with relaxed forepaws and reduced dorsiflexion (Figure 2A), while more severe phenotypes, including hind limb defects, can also be observed (Figure 2C). The *Ark2C* ^−/−^ pups on a 129Sv/Ev genetic background all thin and die during the first 3 postnatal weeks. Postmortem examination revealed dehydration, an empty stomach and gut, and mild cyanosis, suggesting inefficient feeding and breathing.

**Figure 3:**
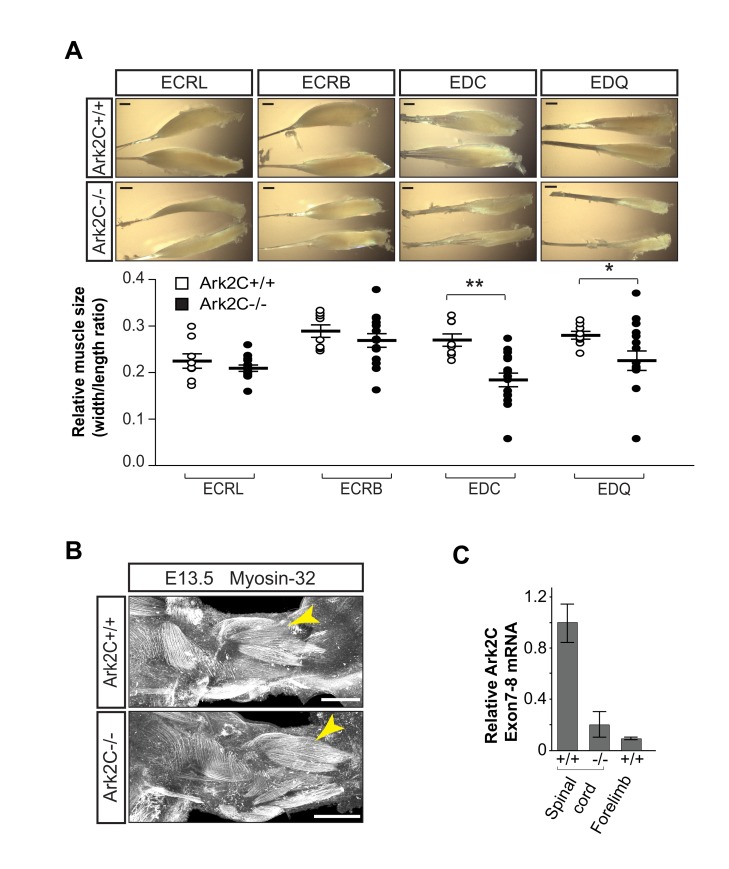
Muscles develop normally but atrophy in adult stages in the absence of Ark2C. (A) Extensor muscles from *wt* and *Ark2C* ^−/−^ adult mice. ECRL, extensor carpi radialis longus; ECRB, extensor carpi radialis brevis; EDC, extensor digitorum communis; EDQ, extensor digiti quinti; R, right limb; L, left limb; scale bars = 1 mm. Dot plot showing the ratio of width/length for the muscles from 4 *wt* and 8 *Ark2C* ^−/−^ mice. Error bars represent ±SEM; * *p*<0.05; ** *p*<0.01. (B) Whole-mount immunofluorescence (IF) showing developing forelimb extensor muscle at E13.5; yellow arrowhead, extensor carpi and digiti muscles; scale bars = 250 µm. (C) Quantitative RT-PCR showing that *Ark2C* is not expressed in E12.5 forelimb (*n* = 3 *wt* embryos) as compared to spinal cord expression in *wt* (positive control) and *Ark2C* ^−/−^ (negative control) embryos. Transcripts measured from exon7–8 including the NRG-TIER and RING domains. Error bars represent ±SD.

In outbred/mixed backgrounds, a small percentage (around 5%) of null animals survive past weaning age. These mice also display mild hypoxia as shown by an increase of blood plasma lactate (mean of 6.11 mM versus 8.08 mM, 0.05>*p*>0.01; Figure 2D) confirming inefficient breathing. As observed in newborns, *Ark2C* ^−/−^ mice examined at P21 or 3–6 months are unable to extend and spread forepaw digits (shown by the absence of clear toes in pawprints in Figure 2E). Measurements of toe prints indicate that this is a significant trait of *Ark2C* ^−/−^ mice (6.5% of footprints have clear toes compared to 100% of *wt* littermates prints, *p*<0.01; Figure 2F). In addition, while the maximum stride width and length of *Ark2C* ^−/−^ animals are normal, a reduction in mean foot length due to reduced toe extension was observed (6.2 mm compared to 8.06 mm, *p*<0.01; Figure 2G and H). Furthermore, these animals exhibit atrophy in the forelimb muscles that control forepaw and digit movement (Figure 3A). As P0 mice display relaxed paws prior to the adult phenotype, developing forelimb muscles were examined in *Ark2C* ^−/−^ embryos (Figure 3B), however no abnormalities that could account for the observed muscle atrophy were observed. X-gal staining showed that *Ark2C* expression is restricted in the nervous system, including the entire spinal cord throughout development and in the adult (Figure 1F–H and unpublished data). Furthermore, quantitative RT-PCR confirmed absence of *Ark2C* expression in *wt* embryonic (E12.5) forelimb RNA (Figure 3C). Together these observations indicate that the defect in *Ark2C* ^−/−^ mice is of neuronal origin and that Ark2C is involved in neuromuscular connectivity. As the majority of the homozygous mice thin and die before weaning, it is unlikely that this function is limited to motor innervation of the forelimb.

### Ark2C Is Required for Efficient Motor Axon Extension in the Dorsal Forelimb

**Figure 4:**
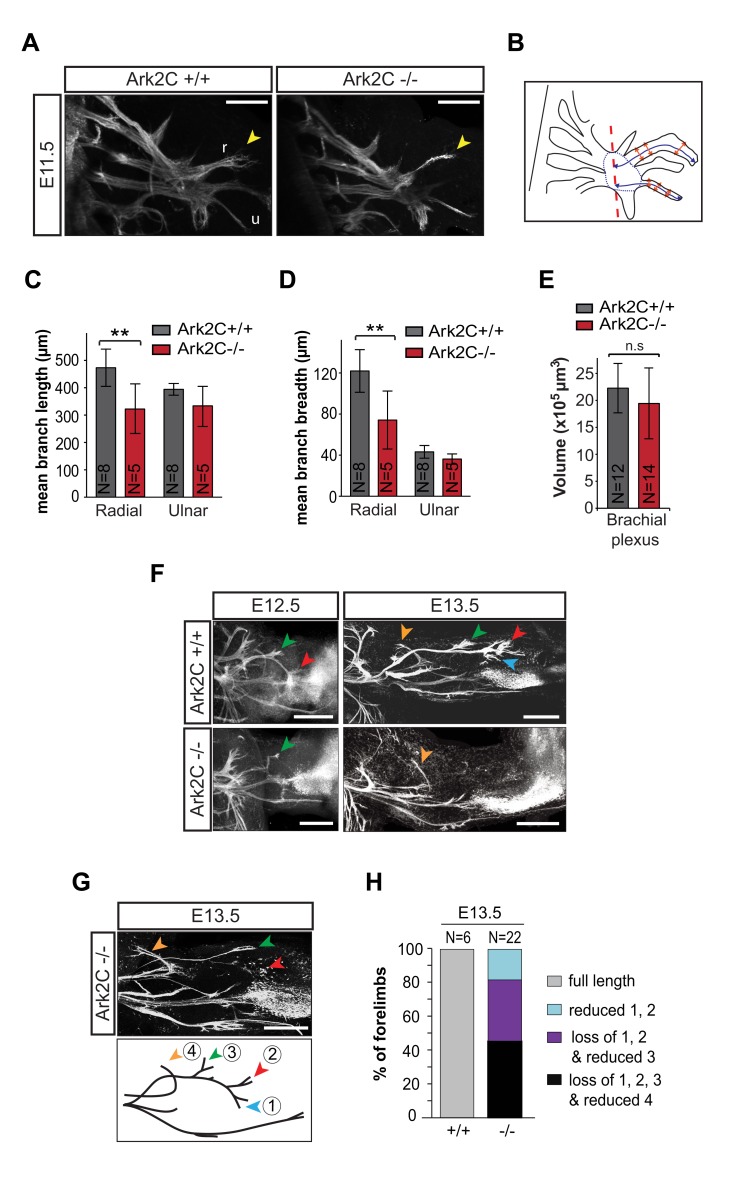
Reduced motor neuron axon growth is observed in the forelimb in the absence of Ark2C. (A) Confocal images from whole-mount IF with anti-GFP showing forelimb innervation in E11.5 HB9-eGFP transgenic embryos. Yellow arrow shows r, radial nerve; u, ulnar nerve; proximal limb to the left; scale bars = 250 µm. (B) Schematic representation of forelimb motor innervation at E11.5. Blue lines indicate measurement of radial and ulnar nerve length from the end of the brachial plexus (dashed red line), orange arrows indicate measurement of nerve width (mean of three measurements), and blue dotted line represents region of volumetric measurements of the brachial plexus. (C–E) Quantification of length and width of the radial and ulnar nerves and volume of the brachial plexus at E11.5; N, number of forelimbs (C–E,H); error bars represent ±SD; ** *p*<0.01; ns, not significant. (F) Confocal images from whole-mount IF with anti-GFP showing forelimb innervation in E12.5–13.5 HB9-eGFP transgenic embryos. Orange, green, red, and blue arrows, as explained in (G), proximal limb to the left; scale bars = 250 µm. (G) An example of an E13.5 *Ark2C* ^−/−^ embryo showing less severe forelimb innervation defects and a schematic representation of the major bifurcation points of the radial nerve at E13.5. Blue (1) and red (2) arrows show branches innervating muscles groups that include EDC and EDQ; green arrow (3) shows branches innervating ECRB and ECRL; orange arrow (4), innervation of more proximal muscles of the dorsal forelimb. (H) Quantification of the severity of the innervation phenotype showing percentage of forelimbs with each classification as indicated at E13.5.

We first analyzed the defective innervation of the forelimb by introducing into Ark2C mutant animals the HB9-eGFP transgene [36], which is expressed specifically in MN. We found that at embryonic day 11.5 (E11.5) *Ark2C* ^−/−^ embryos do not exhibit gross forelimb innervation abnormalities (*n* = 22 forelimbs); motor nerves exit the brachial plexus and project into both the dorsal and ventral forelimb (Figure 4A) [37]. Measurement of these projections found that both the length and width of the more dorsal radial and median nerves are reduced in *Ark2C* ^−/−^ embryos compared to *wt*. No compensatory increase in the size of the more ventral ulnar or thoracodorsal nerves was observed, suggesting that there is no misrouting of axons within the limb bud (Figure 4B–D and Figure S2A–D). Three-dimensional projections of the innervation were also examined for abnormal sprouting of nerves from the brachial plexus, no misrouting was found (*n* = 12 and 14 forelimbs; unpublished data), and the volume of the brachial plexus was not found to differ significantly in size between genotypes (Figure 4E).

As the embryo grows, this phenotype appears to increase in severity and become more specific to nerves innervating the dorsal region of the limb. By E12.5, the radial nerve thins, and axons do not reach the more distal dorsal target muscles in 21 out of 24 *Ark2C* ^−/−^ limbs examined (Figure 4F), while the ventral axon projections (ulnar and median nerves) parallel those of *wt* embryos. Similarly, at E13.5, *Ark2C* ^−/−^ limbs exhibit dorsal muscle innervation deficits to varying degrees with increased severity towards the distal muscles controlling the digits (Figure 4G and 4F; Figure S2G; Movies S1 and S2). We compared confocal images from *wt* and *Ark2C* ^−/−^ forelimbs focusing on the presence and the intensity of the major partition points of the radial nerve. These partitions are clearly visible at E13.5 and correspond to specific forelimb muscle groups (mapped by backfills, T.M. Jessell, personal communication) as shown in the diagram (Figure 4G). The distal partitions (arrowheads 1 and 2, Figure 4G) innervate muscle groups such as the extensor digitorum communis (EDC) and extensor digiti quinti (EDQ), while proximal partitions (arrowheads 3 and 4) innervate muscles including the extensor carpi radialis longus (ECRL) and brevis (ECRB). All *Ark2C* ^−/−^ forelimbs examined exhibit deficits in the distal partitions and more than half also exhibited proximal deficits (Figure 4H). Forelimb motor defects were observed in all (250+) *Ark2C* ^−/−^ embryos and newborns (Figure 2A) analyzed to date independent of genetic background, however a similar phenotype has never been observed in heterozygote siblings (unpublished data).

The above data suggest that Ark2C is not required for the initial axon projection of the radial nerve from the brachial plexus into the dorsal forelimb mesenchyme, but it is essential for further motor axon advancement. The requirement for Ark2C increases as the embryo grows and is most obvious in motor axons that are required to grow the furthest to innervate more distal muscles. While there is thinning of the main radial nerve bundle and its partitions to various extensor muscles, the path taken by the nerve and the positions of characteristic points of bifurcation are not altered in the absence of Ark2C. These observations support a role for Ark2C in axon growth and not in path or target finding. The requirement for Ark2C function differs between nerves and embryonic stages: the radial nerve shows an increasing requirement with age, the median nerve requires Ark2C at E11.5, but this need lessens with age while the ulnar nerve appears unaffected by loss of expression. These differences in requirement for Ark2C between MN are likely to be due to intrinsic differences in specification of the cells, the properties of the peripheral substrate that their axons elongate through, or an interaction between these two factors.

### Ark2C Is Required for Normal Length of the Phrenic Nerve Presynaptic Branches

**Figure 5:**
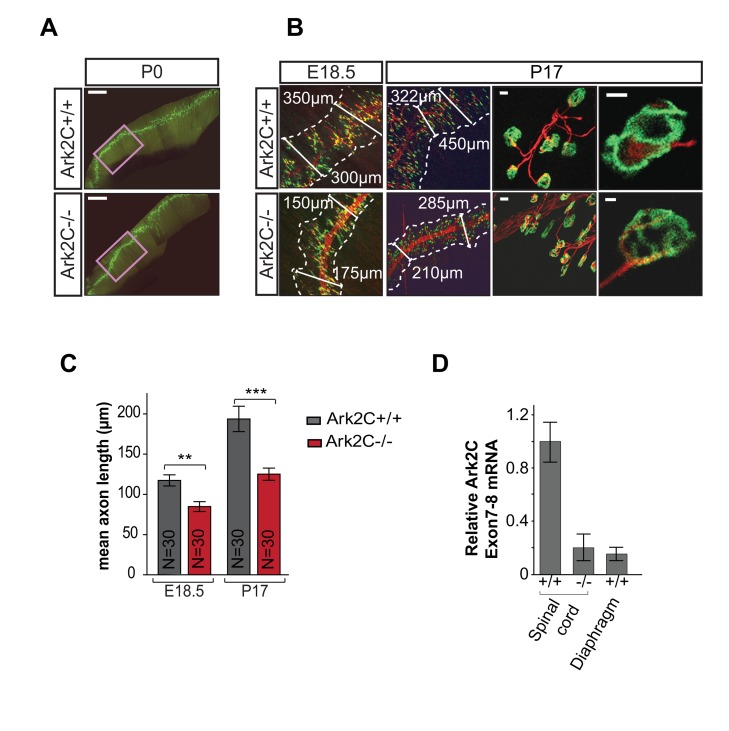
Reduced motor neuron axon growth is also observed in the diaphragm in the absence of Ark2C. (A) Whole-mount IF of diaphragm muscle showing the phrenic nerve and synapses in the entire muscle at P0; purple box shows area analyzed in (B); scale bars = 1 mm. (B) Higher magnification images at E18.5 and P17. α-Bungarotoxin (green), postsynaptic marker; neurofilament and synaptophysin (red), axons and presynaptic terminals; scale bars = 10 µm. (C) Histogram showing the measurements of axon length from the phrenic to the synapses in *wt* and *Ark2C* ^−/−^ diaphragms at E18.5 and P17; N, number of axons. Error bars represent ±SEM; ** *p*<0.01; *** *p*<0.001. (D) *Ark2C* expression in *wt* and *Ark2C* ^−/−^ embryonic (E12.5) spinal cord and *wt* (E19.5, *n* = 2) diaphragm muscle measured by quantitative RT-PCR as in Figure 3C.

To examine the extent of the requirement for Ark2C in MN projections, we analyzed the innervation of the diaphragm by the phrenic nerve, as this may be involved in the milder hypoxia phenotype observed in *Ark2C* ^−/−^ animals (Figure 2D). In both *Ark2C* ^−/−^ embryos and surviving adults, the phrenic nerve forms synapses throughout the length of the diaphragm muscle (*n* = 25; Figure 5A–B), however the synapses are clustered together around the intramuscular nerve fiber appearing to be at a greater density than in the *wt* (Figure 5B). This increased density could reflect either an increase in the number of synapses formed or their distance from one another. Therefore, the number of synapses per mm of phrenic nerve and the width of the synaptic band were measured. By P17 *Ark2C* ^−/−^ animals are significantly smaller than their littermates, affecting the size of muscles and nerves, however at E18.5 *Ark2C* ^−/−^ embryos are of normal size. At this age loss of Ark2C expression produced a slight increase in number of synapses (351.0 synapses/mm of axon in *wt* and 373.5 synapses/mm in *Ark2C* ^−/−^) within the diaphragm; in addition, comparison of a single region of *wt* and *Ark2C* ^−/−^ diaphragms (Figure 5A) revealed that the width of the synaptic band is reduced in *Ark2C* ^−/−^ diaphragms (Figure 5B), further increasing the density of synapses. The length of individual axons from the phrenic nerve to the synapse within the synaptic band was also reduced in both embryo and pup (mean length at E18, 117.30 µm and 84.75 µm, *p*<0.01; mean length at P17, 193.61 µm and 125.02 µm, *p*<0.01; Figure 5B and C), suggesting that loss of Ark2C affects the extent rather than the directionality of terminal branch growth in the embryonic diaphragm. Quantitative RT-PCR showed that similar to the developing limb, Ark2C is not expressed in the diaphragm muscle at E19.5, the stage when the innervation phenotype becomes apparent (Figure 5D). This result supports a neuronal origin of the diaphragm innervation defect.

The synapses in the *Ark2C* ^−/−^ P17 diaphragm have a pretzel-like morphology, suggesting normal activity-dependent maturation occurs postnatally. However, from the above analysis we cannot estimate the impact of short presynaptic motor branches on the function of the neuromuscular synapse and diaphragm muscle. It is possible that in the absence of Ark2C similar defects exist in MN that innervate additional muscles involved in breathing and that the observed hypoxia phenotype is cumulative from a number of innervation defects.

The analysis of the forelimb and diaphragm innervations in *Ark2C* ^−/−^ embryos and adult mice shows that Ark2C functions primarily during development and growth and is required for efficient MN neuromuscular connectivity within target muscles. The enhancement of connectivity by Arkadia2C ranges from the advancement of major nerves such as the radial nerve through the developing limb bud to a more subtle growth of the presynaptic branches of the phrenic after entering the diaphragm muscle.

### Lateral Motor Column Specification Does Not Depend on Ark2C

**Figure 6:**
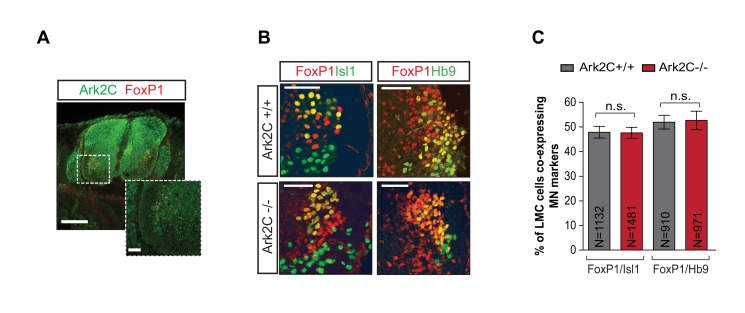
Motor neuron specification is normal in the absence of Ark2C expression. (A) IF showing Ark2C expression (β-gal, green) and FoxP1 (red) in spinal cord at E13.5; scale bars = 250 µm and 50 µm for the magnified insert. (B) IF showing motor pool marker expression in spinal cord at E13.5. (C) Percentages showing the number of FoxP1-expressing cells (LMC) that also express Isl1 (LMC_m_) or high Hb9 (LMC_l_) are shown in the histogram; N, number of cells; n.s., not significant; scale bars = 50 µm.

Next we investigated the cause of the variable requirement of Ark2C between MN that innervate the dorsal and ventral forelimb at E12–13.5. In the spinal cord, the lateral and medial divisions of the Lateral Motor Column, (LMC_l_ and LMC_m_ respectively) consist of adjacent motor pools that innervate the dorsal and ventral limb, respectively [37]–[39]. Unique combinations of transcription factors mark the various motor pools (Figure S3A) [38],[40]–[43]. We excluded the possibility that the specificity of the innervation defect in the dorsal limb is due to restricted Ark2C expression only in the lateral LMC because Ark2C (β-gal) was found to be present in both lateral and medial LMC pools (expressing FoxP1) in E13.5 *Ark2C* ^+/−^ brachial spinal cord (Figure 6A).

Furthermore, we examined whether loss of Ark2C either specifically causes loss of LMC_l_ neurons or causes misspecification to an LMC_m_ identity. The motor pools were examined at E13.5 (*n* = 13 embryos from each genotype; Figure 6B and C and Figure S3B and C) as there is a substantial loss of dorsal innervation at this age (Figure 4F). LMC_m_ cells co-express FoxP1 and Isl1, while LMC_l_ cells express FoxP1 and high levels of Hb9 in their nuclei. The number of cells in these domains was found to be the same in *wt* and *Ark2C* ^−/−^ embryos (Figure 6C; 47.8% compared to 47.6%, *p* = 0.93 and 51.9% compared to 52.6%, *p* = 0.89, respectively). Therefore, dorsal-ventral MN misspecification cannot account for the substantial loss of radial nerve projections to the forelimb in *Ark2C* ^−/−^ embryos.

At E11.5 when the limb innervation defect is initiated in *Ark2C* ^−/−^ embryos, MN have not formed synapses with other distant neurons [44],[45], implying that Ark2C functions either within the LMC itself or in adjacent cells. Secretion of a signal by adjacent cells that is required to a greater degree by LMC_l_ than LMC_m_ neurons would allow these cells to indirectly affect predominantly the LMC_l_ axonal projection in the limb. Alternatively, as the defect becomes more localized to dorsal innervation between E11.5 and 13.5, Ark2C may be required within the LMC neurons for the interpretation of a guidance signal from the periphery. This putative signal could be unevenly distributed between the dorsal and ventral limb, creating a greater requirement for Ark2C in LMC_l_ than LMC_m_. Both hypotheses implicate a signal involved in axon projection.

### Ark2C Via Its RING Domain Enhances BMP-Smad1/5/8 Signaling in the Spinal Cord

**Figure 7:**
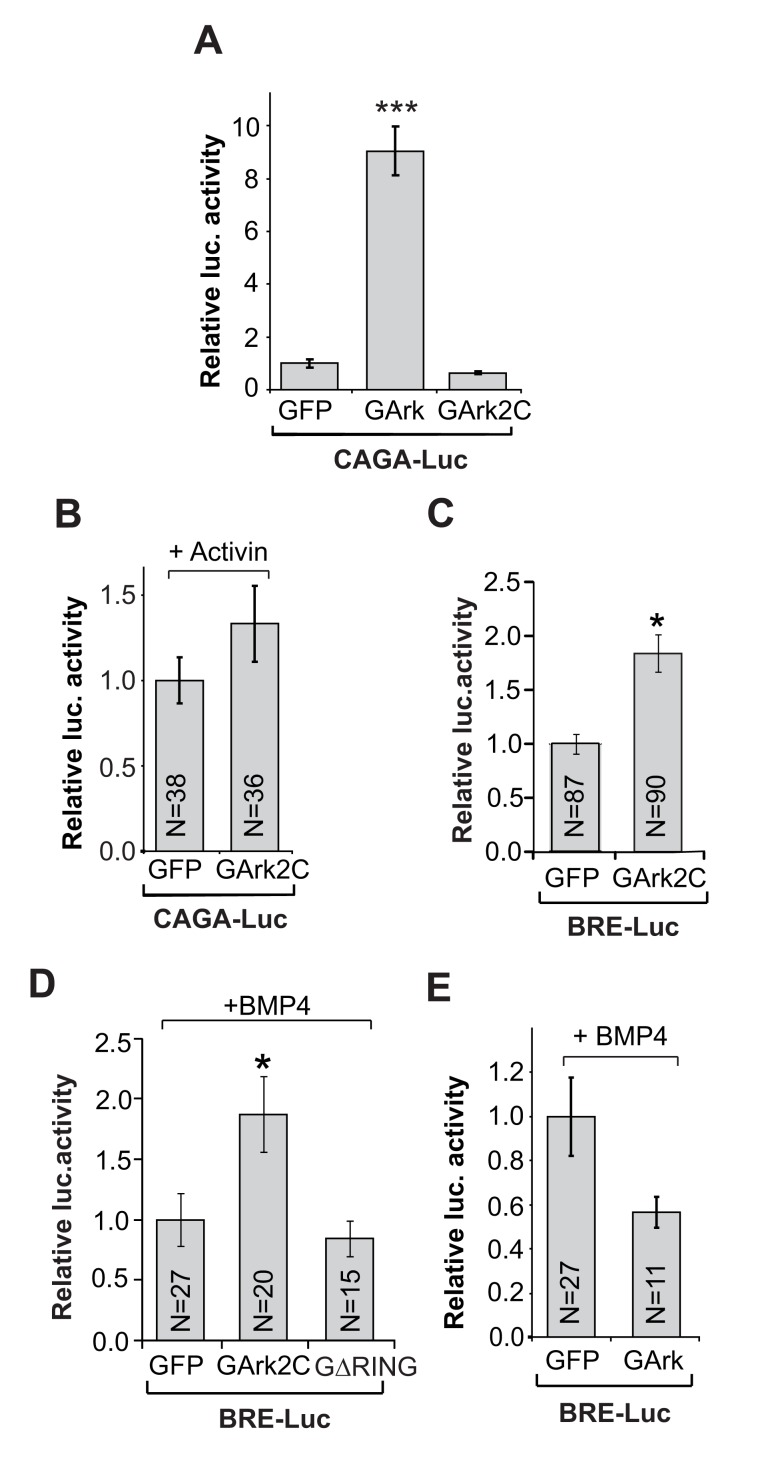
Ark2C enhances the transcriptional responses of Smad1/5/8. (A) CAGA12-luciferase activity in Arkadia-null mouse embryonic fibroblasts transfected with plasmids expressing GFP, GFP-Ark (GArk), or GFP-Ark2C (GArk2C). Error bars represent ±SEM; *N* = 4 for all; *** *p*<0.001. (B) CAGA12-luciferase activity in chick neural tubes electroporated with GFP or GArk2C and Activin. Error bars represent ±SEM. N, number of embryos. (C–E) BRE-luciferase activity in chick neural tube electroporated with plasmids expressing GFP, GArk, GArk2C, GFP-hArk2CΔRING (GΔRING), or BMP4. Error bars represent ±SEM; N, number of embryos; * *p*<0.05.

The above analysis of the *Ark2C* ^−/−^ forelimb innervation defect suggests that Ark2C regulates either the production of a signal by spinal cord cells adjacent to MN, or the response of MN to a signal from the periphery. In both cases, the signal promotes or sustains axon extension. To find the signaling pathway that is affected by Ark2C, we examined its homology to other proteins. The C-terminal domain of Ark2C contains its most conserved domain, the RING, which is highly homologous to that of Arkadia (Figure 1B). Arkadia enhances the responses downstream of the Nodal-TGF-β pathway, transduced by the Smad2/3 effectors by mediating the ubiquitin/proteasome degradation of negative regulators of the pathway [33],[35]. To assess the similarity of Ark2C to Arkadia we used Arkadia-null mouse ES cells and embryonic fibroblasts to measure transcriptional activation of a Smad2/3-dependent luciferase reporter by Ark2C. In these null cells, expression of Arkadia (GFP tagged; GArk) can restore high levels of Smad2/3-dependent transcription, but Ark2C (GFP tagged; GAkd2C) cannot (Figure 7A and unpublished data). Moreover, Ark2C cannot enhance the Smad2/3 reporter activity in a neuronal context after electroporation in the embryonic chick spinal cord (Figure 7B). However, Ark2C enhanced by 2-fold the activity of a BMP-Smad1/5/8 responsive (BRE) luciferase reporter in chick spinal cord (Figure 7C and D). As an enzymatically inactive mutant form of Ark2C (GΔRING) did not increase BRE-luciferase activity above the level of the control GFP (Figure 7D), we concluded that this enhancement depends on the ligase activity of the Ark2C RING domain. Furthermore, Arkadia (GArk) could not activate the BRE-luciferase reporter even in the presence of BMP4 (Figure 7E), confirming that Arkadia enhances specifically the Smad2/3 branch of TGF-β signaling. Therefore, Ark2C not only shares homology with the Arkadia RING domain but also functions in a similar way. However, while Arkadia enhances the Smad2/3 transcriptional responses, Ark2C specifically enhances Smad1/5/8 via its RING/ubiquitin ligase domain.

Given that the chick spinal cord expresses endogenous Ark2C (unpublished data), the 2-fold enhancement that we observed upon overexpression is significant. Furthermore, Smad1/5/8 effectors regulate several endogenous target genes downstream of BMP ligands, suggesting that the sum of transcriptional enhancement by Ark2C could have a phenotypic impact.

### Ark2C Derepresses pSmad1/5/8 Signaling by Interacting With and Degrading Negative Regulators

**Figure 8:**
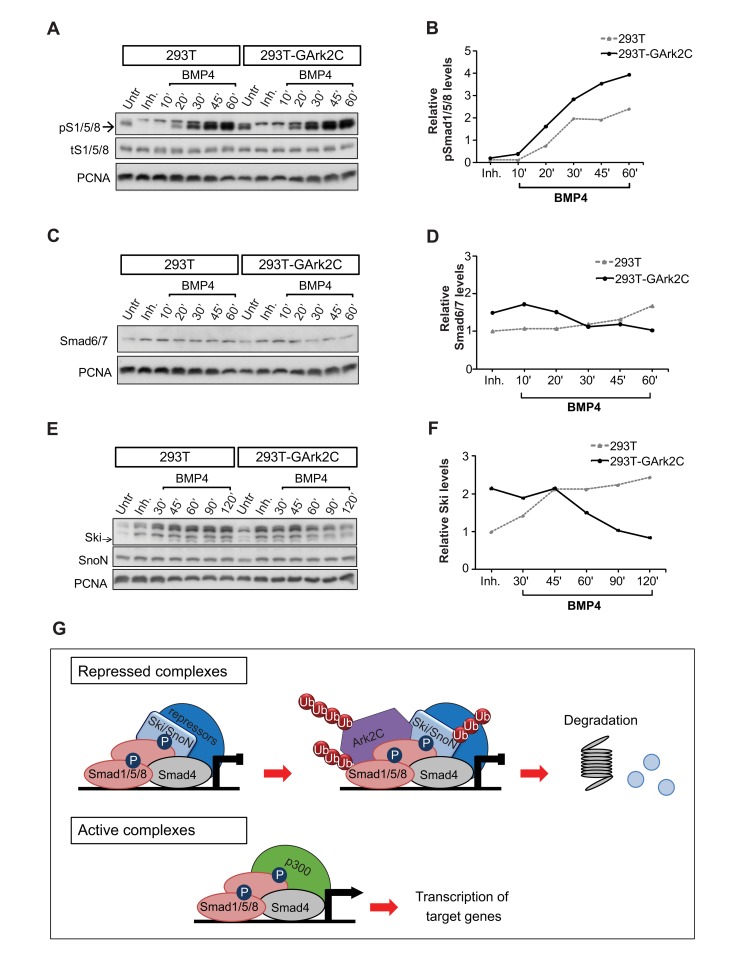
Ark2C enhances BMP signaling by degrading negative regulators of the pathway. Immunoblots (IB) showing pSmad1/5/8 and total Smad1/5/8 (tS1/5/8) (A), Smad6/7 (C), and Ski, SnoN (E) in 293T and stable clones of 293T-GArk2C cells treated with dorsomorphin (Inh.) or BMP4 in serum-free medium. Protein levels of pSmad1/5/8, Smad6/7, and Ski were quantified, normalized to PCNA, and the relative protein levels are shown in arbitrary units in the histographs (B, D, and F, respectively). Untr, untreated cells cultured in 10% FBS; arrows indicate the bands used for quantification. (G) Model for derepression of transcription by Ark2C. Upon BMP stimulation, complexes form between pS1/5/8 and Smad4, and the negative regulators Ski/SnoN repress these complexes on gene promoters. Repressed complexes are degraded by Ark2C (top), allowing freshly activated Smads to enhance transcription (bottom). The cycle is repeated as the Ski/SnoN presence is maintained and up-regulated by pSmad1/5/8.

To investigate Ark2C molecular function, we first studied the Smad1/5/8 activation profile under BMP4 stimulation. For this we used HEK293T cells (293T) that do not normally express endogenous Ark2C, and 293T stably expressing low levels of GArk2C or transiently transfected with FLAG-Ark2N or FLAG-Ark2C (Figure 8A and B and Figure S4A and B). Smad1/5/8 were activated earlier in the presence of GArk2C or FLAG-Ark2C but not with FLAG-Ark2N (Figure S4A and B), indicating that faster effector phosphorylation is a property of Ark2C. Arkadia mediates the ubiquitin/proteasome degradation of inhibitory Smad6/7 [31]; we therefore examined the levels of these negative regulators in the above experiment during BMP4 stimulation. At 1 hour, there was a reduction in the levels of Smad6/7 in the presence of Ark2C (Figure 8C and D and Figure S4C and D), suggesting that these inhibitory Smads are also substrates of Ark2C. Therefore, the two Arkadia proteins act in a similar manner. Smad6/7 inhibits signaling by mediating the degradation of the receptors and by interfering with the phosphorylation of pSmad1/5/8. Although Ark2C degrades Smad6/7 (Figure 8C and 8D), its presence does not have a profound effect on the steady state of pSmad1/5/8 levels (Figure S4G and S4H). The initial high levels of pSmad1/5/8 in the presence of Ark2C are not maintained most likely due to an autoregulatory transcriptional loop in which Smad6/7 are up-regulated by pSmads [46]. According to this model, when pSmads levels are increased Smad6/7 levels are also up-regulated, leading to a reduction of receptors and Smad1/5/8 phosphorylation. It appears that this feedback loop is capable of correcting intracellular signal fluctuations and absorbs the initial effect caused by the presence of Ark2C. Therefore, the reduction of Smad6/7 by Ark2C at the initial phase of signal activation is unlikely to be the major underlying cause of the null phenotype and cannot account for the 2-fold transcriptional enhancement that we observe 24 h after electroporation of Ark2C in chick spinal cord (Figure 7C and 7D).

The co-repressors Ski/SnoN are degraded by Arkadia, when they are in a complex with pSmad2/3 [32],[33],[44]. We therefore examined if they are also degraded by Ark2C. During the first 2 h under BMP4 stimulation, the protein levels of the co-repressor Ski were found to be reduced (Figure 8E and 8F) in a reproducible manner (Figure S4E and S4F). The levels of the Ski-like factor, SnoN, remained unchanged (Figure 8E), suggesting that in the 293T cell context Ark2C shows a preference for Ski, which is consistent with similar data on Arkadia [47].

The co-repressors Ski/SnoN form complexes with pSmads on the promoters and repress transcription by recruiting histone deacetylases. A simple reduction in the overall levels of nuclear Ski/SnoN cannot reverse this repressive mark from the promoters, and physical removal of these repressed complexes is required. Recurrent clearance of the promoters from this repression allows fresh and unrepressed pSmads to bind and recruit p300 and other co-activators to re-initiate transcription throughout signal stimulation (Figure 8G). Ark2C and Arkadia appear to do exactly this: degrade Ski/SnoN repressors specifically when they are interacting with and repressing pSmads. This function results in enhancement of the transcriptional responses from pSmad target gene promoters in the presence of Arkadia proteins and can account for a continuous requirement of Ark2C and most likely for the 2-fold enhancement of the BRE-reporter transcription observed 24 h after electroporation of Ark2C in chick spinal cord (Figure 7C and 7D).

The amount of Smad6/7 degraded by Ark2C appears low because there is rapid protein recovery achieved due to the resultant increase of signaling by pSmads, which transcriptionally up-regulate Smad6/7 in an autoregulatory loop. The reduction of Ski protein also appears small as Ski degradation by Ark2C is restricted to the fraction that is actively repressing pSmads potentially when they are associated with the promoters of target genes. However, the removal of this likely quite small fraction of total Ski is expected to cause profound derepression of promoters and enhancement of gene transcription.

**Figure 9:**
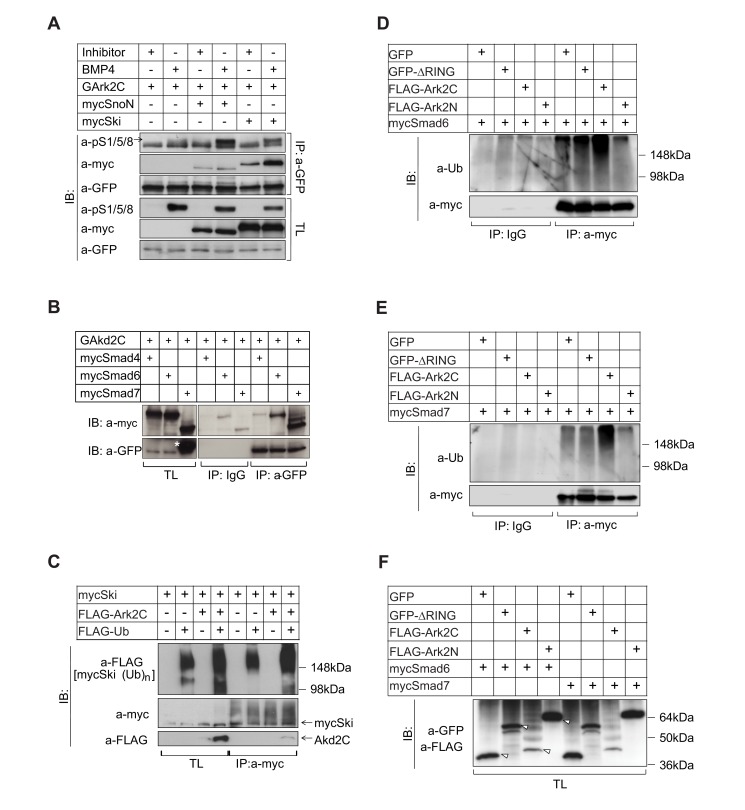
Ark2C interacts with and ubiquitinates negative regulators via its RING domain. (A) Immunoprecipitation (IP) in 293T-GArk2C cells after 1 h treatment with dorsomorphin (Inhibitor) or BMP4 in serum-free medium and in the presence of MG132. TL, total lysate; arrow, pSmad1/5/8 specific band. (B) IP in 293T-GArk2C cells showing interactions with mycSmad6 and mycSmad7 but not with mycSmad4. The same membrane was used for IB with a-GFP and leftover signal from mycSmad7 is indicated by *. (C) IP showing the ubiquitination of mycSki in 293T cells transfected as indicated. (D–E) IP showing the ubiquitination of mycSmad6 and mycSmad7 in 293T cells transfected as indicated. The expression of the proteins in the total lysates is shown in (F). Arrowheads indicate specific bands.

We investigated how directly Ark2C interacts with the above candidate substrates using immunoprecipitation (IP) with GFP antibody in HEK293T cells stably expressing low levels of GArk2C. These experiments showed that Ark2C interacts with endogenous pSmad1/5/8 (Figure 9A), with the repressors SnoN and Ski (Figure 9A), and with the inhibitory Smad6/7 (Figure 9B), but not with endogenous pSmad2/3 (Figure S5A). Interestingly the overexpression of either SnoN or Ski increased the fraction of endogenous pSmad1/5/8 interacting with Ark2C (Figure 9A), suggesting that Ark2C interacts with complexes between repressors and effectors rather than the individual proteins. Furthermore, the presence of Ark2C, and not that of Ark2N or a RING-less version of Ark2C, increased polyubiquitination of the above proteins (Figure 9C–F), implying that these are the substrates of Ark2C ubiquitin ligase activity. Collectively the above results confirm that the substrates of Arkadia and Ark2C comprise the same set of negative regulators, but that Ark2C mediates their degradation in the presence of pSmad1/5/8.

**Figure 10:**
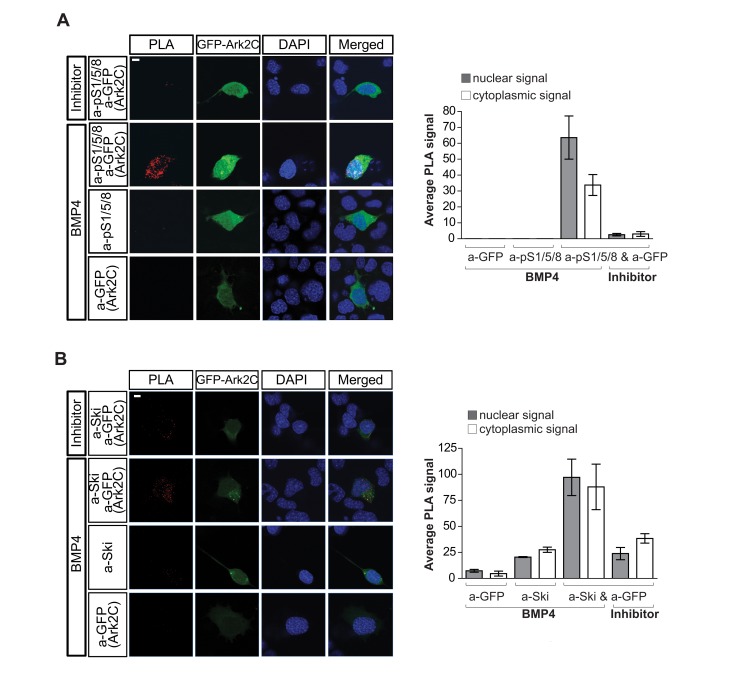
PLA showing that Ark2C interacts with components of the BMP pathway *in situ*. Confocal images of Proximity Ligation Assay (PLA) performed in NSC-34 cells transfected with GFP-Ark2C and treated for 1 h as indicated showing interaction of Ark2C with endogenous pSmad1/5/8 (A) or Ski (B). The use of a single antibody served as negative control. PLA signal from transfected cells (3–5) was quantified, and the results are shown in the histogram on the right. Red spots, PLA signal; blue, DAPI-nucleus; scale bars = 10 µm.

We next visualized the interaction of Ark2C with endogenous protein substrates within the cell using proximity ligation assay (PLA), a technique that detects protein–protein interactions *in situ*. This showed that Ark2C (FLAG-Ark2C) is in close proximity with endogenous pSmad1/5/8 and Ski in the nucleus of 293T cells in the presence of BMP4 stimulation, while there is no formation of complexes in the presence of BMP inhibitor (Figure S5B). Furthermore, the above molecular interactions were confirmed by PLA in the MN-like environment of the cell line NSC-34 [48], where endogenous Ark2C is expressed (Figure S6A). Transfected GArk2C interacts with endogenous pSmad1/5/8 (Figure 10A) and also with Ski (Figure 10B) only in the presence of BMP4 stimulation. Collectively, the above data position Ark2C specifically downstream of the BMP-Smad1/5/8 branch of TGF-β signaling and are consistent with the notion that it functions by derepression and ubiquitin-mediated degradation of negative regulators of the pathway.

### BMP-Smad Signaling Is Involved in Motor Axon Elongation

**Figure 11:**
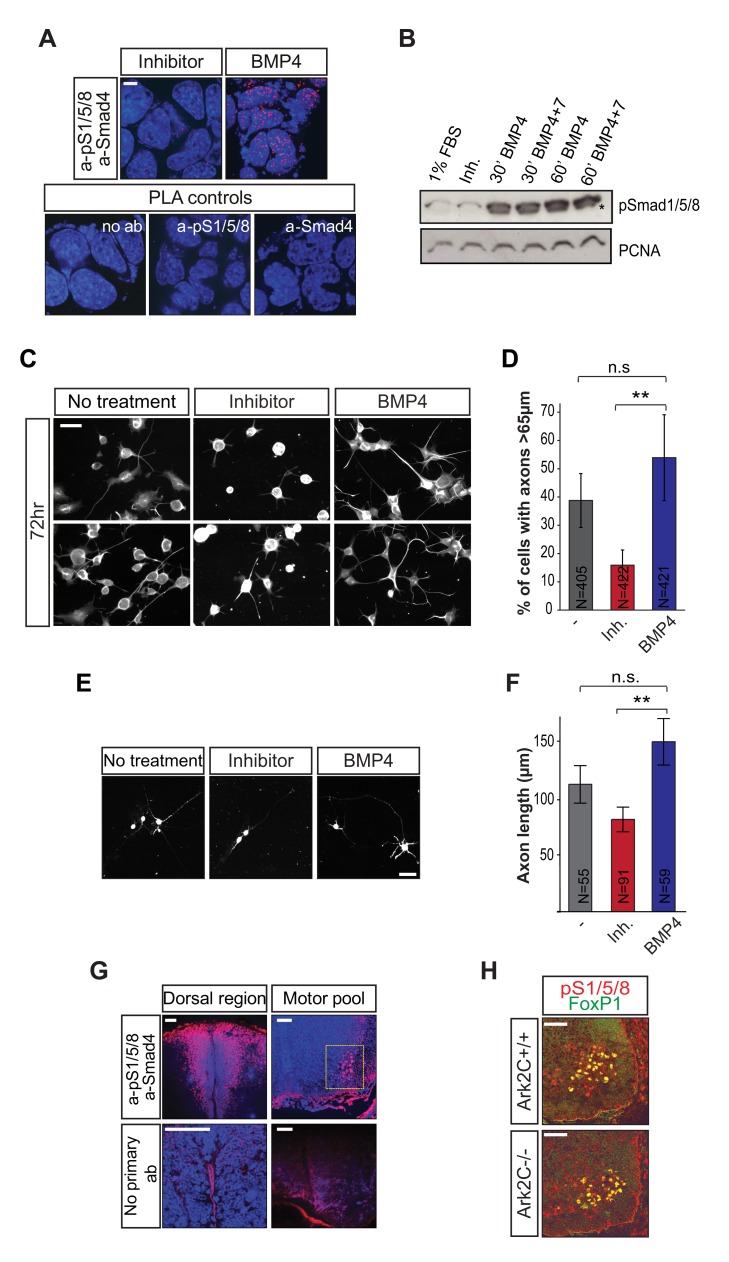
BMP-Smad signaling is present in motor pools and enhances MN axon growth in culture. (A) Confocal images from PLA in NSC-34 cells showing pSmad1/5/8-Smad4 complexes after treatment with dorsomorphin (Inhibitor) or BMP4. The omission of the primary antibodies or the use of a single antibody served as negative controls. Red spots, PLA signal; blue, DAPI-nucleus; scale bars = 10 µm. (B) IB showing pSmad1/5/8 in NSC-34 cells treated with dorsomorphin (Inh) or BMP4 and 7. PCNA was used as loading control; * specific band. (C–D) Representative IF confocal images showing neurofilament in NSC-34 cells after 72 h of treatment with 1% FBS+dorsomorphin (Inhibitor) or 1% FBS+BMP4. Nontreated cells were maintained in 1% FBS. Cells with long axons (as measured in Figure S6D) were counted and percentage to the total number of cells is shown in a histograph. Scale bars = 200 µm; N, number of cells; error bars represent ±SD between experiments and reflect counts in different slides; ** *p*<0.01; n.s., not significant. (E–F) Confocal images of primary MN derived from E13.5 HB9-eGFP embryos stained with anti-GFP antibody after treatment for 72 h with dorsomorphin (Inhibitor) or BMP4. The length of the axons was measured and shown in the histograph. ** *p*<0.01; N, number of cells; n.s., not significant. (G) Confocal images of brachial spinal cord vibrotome sections after PLA detecting pSmad1/5/8-Smad4 complexes in the spinal cord at E13.5; lower panels, the omission of the primary antibodies served as negative control. Red spots, PLA signal; blue, DAPI-nucleus; scale bars = 50 µm. (H) IF showing pSmad1/5/8 and FoxP1 in brachial motor pools of E13.5 *wt* and *Ark2C* ^−/−^ embryos. Scale bars = 50 µm.

To address the role of BMP in MN axon advancement we first used NSC-34 cells [49], which share many of the morphological and physiological properties of motor neurons; within 24 h under 1% Fetal Bovine Serum (FBS), NSC-34 cells extend axons and express MN markers (Figure S6B). NSC-34 respond to BMP treatment, as shown by immunoblot (IB) for pSmad1/5/8 and PLA for pSmad1/5/8–Smad4 complexes, which form only in the presence of BMP stimulation (Figure 11A and B). We assayed the effect of BMP4 or inhibitor on axon growth and elongation by measuring axon length after 48 and 72 h of treatment. At 48 h the number of cells with long axons (>65 µm from the center of the cell; method shown in Figure S6D) was not significantly different between treatments (BMP:150/400 and Inh:189/419, Figure S6C). However at 72 h (Figure 11C–D), the number of cells with long axons was reduced by 3.4-fold on treatment with inhibitor compared to BMP4 or by 2.4-fold when compared to the untreated control (Inh, 67/422; BMP4, 227/421; Untr, 157/405 difference between treatments, *p*<0.01). The axon growth in the “untreated” cells is likely due to the fact that all cells were maintained in 1% FBS, which normally contains BMP ligands and activates pSmad1/5/8 to a small degree (unpublished data). The delayed effect (3 d) of the inhibitor on axon growth suggests that BMP signaling in NSC-34 cells is required for motor axon elongation rather than MN differentiation or initiation of axon growth, and that it involves nuclear transcription and Smads. The experiment was repeated with primary MN from HB9-GFP embryos (E13.5) and showed also that BMP enhances, while inhibitor delays, motor axon advancement in culture (Figure 11E and F). It should be stated that the primary MN were allowed to initiate axon growth before being subjected to the treatments so that we could assess the effect on axon elongation and not on axon regrowth.

To address whether BMP signaling also plays a role in motor axon elongation in vivo, we examined signaling activation in motor pools using PLA. PSmad1/5/8-Smad4 complexes were detected in the nuclei of spinal cord roof plate cells, where BMP ligands are abundant, and also in the brachial ventral horn containing the LMC (Figure 11G; series of spinal cord sections in *n* = 3 E11, E12, and E13 embryos). Co-staining for pSmad1/5/8 and FoxP1 showed that signaling occurs in all LMC neurons (Figure 11H; multiple sections from *n* = 6 *wt* and 6 null embryos). There was no obvious reduction of pSmad1/5/8 in the LMC/FoxP1 domain of *Ark2C* ^−/−^ embryos (Figure 11H), which is consistent with the molecular findings that Ark2C enhances the downstream transcriptional responses by derepressing promoters rather than by changing the steady state level of pSmads. Collectively the above results support an involvement of BMP-Smad signaling in MN axon projections. BMP ligands are abundant in the periphery (i.e., limb) and the dorsal spinal cord but not in the motor pools [50],[51]. Therefore, it is likely that the critical role of Ark2C is to enhance the response of ventral spinal cord cells (i.e., MN) to BMP signals originating at a distance.

### The Ark2C Phenotype Is Mediated at Least in Part by BMP Signaling

Several BMP ligands are present in the developing limb [52]; however, their expression is dynamic, indicating a spatio-temporal variation in signal availability in the periphery (Figure S7). It is therefore possible that Ark2C functions within MN to maintain signaling responses when ligand availability or signaling ability fluctuates. We used genetics to test this hypothesis, taking advantage of the fact that loss of only one allele of *Ark2C* (*Ark2C* ^+/−^) does not lead to any forelimb posture and movement defects. We examined how forelimb innervation is affected when we reduce BMP signaling genetically.

**Figure 12:**
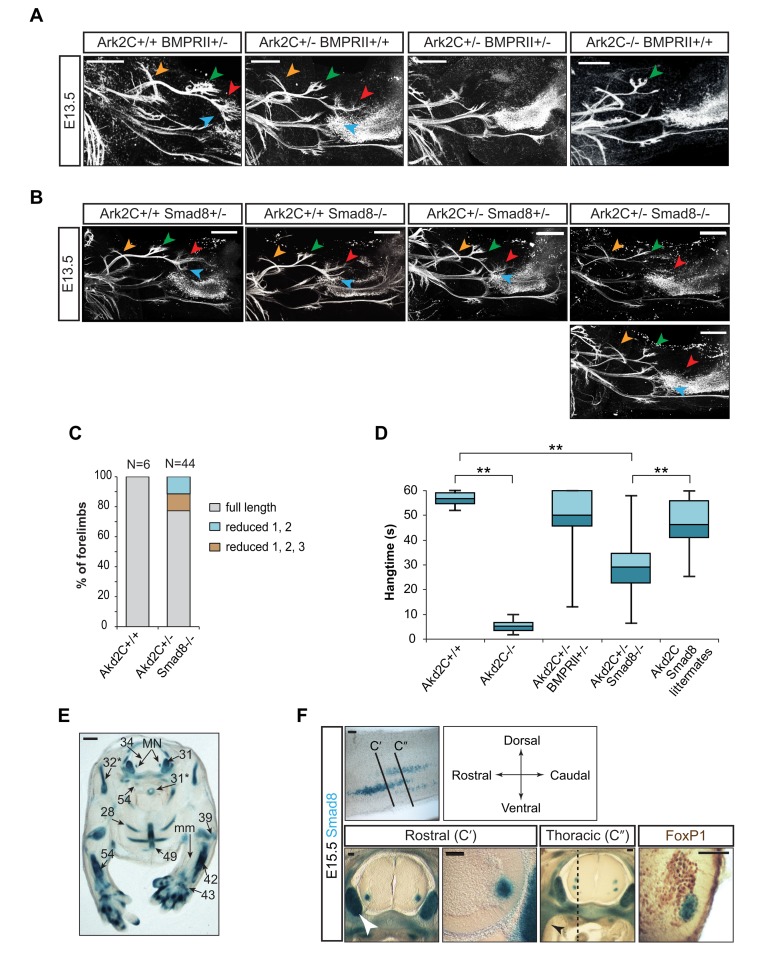
Genetic reduction of BMP signaling and Ark2C in motor pools reproduces the dorsal forelimb innervation defects. (A–B) Confocal images of whole-mount IF with anti-GFP showing motor neuron innervation of the forelimb in HB9-eGFP embryos from crosses between *Ark2C* ^+/−^;*BMPRII* ^+/−^ and *Ark2C* ^+/−^ (A) and between *Ark2C* ^+/−^;*Smad8* ^+/−^ and *Smad8* ^−/−^ (B). Arrows as in Figures 4F and S7A; scale bars = 250 µm. (C) Quantification of the innervation at E13.5 shown as percentages of forelimbs, each phenotypic classification as described; N, number of forelimbs. (D) The population spread of average hang time in the cage lid test for each genotype. Ark2C Smad8 littermates, mix of *Ark2C^+/+^*:*Smad8* ^+/−^, *Ark2C^+/+^*:*Smad8* ^−/−^. *Ark2C* ^+/−^:*Smad8* ^+/−^. ** *p*<0.01. (E) Transverse section of E15.5 *Smad8* ^+/−^ embryo at brachial level after X-gal staining showing Smad8 expression in bone, cartilage, DRG, and motor pools. Numbers as in “The Atlas of Mouse Development” [75] 31*, 32*, 54* on pp 266–267 and 28, 31, 34, 39, 42, 43, 49, 54 on pp 268–269; mm, muscle mass; MN, motor neurons. (F) Sagittal and transverse (C′ and C″) vibrotome sections of the spinal cord from a *Smad8* ^+/−^ embryo stained with X-gal showing Smad8 expression in motor pools or co-stained with antibody against FoxP1. White arrowhead, DRG; blue, cytoplasmic X-gal stain of Smad8 expression; brown, nuclear FoxP1 expression; sagittal section, dorsal towards top; scale bars = 100 µm. Dotted line indicates position of sagittal section in the first panel; first rib is indicated by black arrowhead.

BMP type II receptor (BMPRII) is an essential and unique core component of BMP signaling. *BmprII* null mice die early during gastrulation, but mice with one allele breed successfully and are normal [53]. Analysis of the offspring from crosses between *Ark2C* ^+/−^;*BmprII* ^+/−^ and *Ark2C* ^+/−^ mice showed that fewer than expected *Ark2C* ^+/−^;*BmprII* ^+/−^ double heterozygotes were born (genotyped within 24 h after birth). However, all those born survived to adulthood (Table S1A). MN development was analyzed via expression of the HB9-eGFP transgene in embryos from the same cross. This showed that single heterozygous mice (*Ark2C* ^+/+^;*BmprII* ^+/−^) have robust innervation of the dorsal limb, while a subset of E13.5 double heterozygotes (*n* = 2 out of 6 examined) exhibit innervation defects in the dorsal forelimb similar to the severely affected *Ark2C* ^−/−^ embryos (Figure 12A).

We also assessed limb weakness in *Ark2C* ^−/−^ mice and compound mutants by measuring the time that the mouse could suspend itself from a cage lid (Figure 12D). This test requires repeated gripping and releasing of the bars and therefore unlike standard grip strength tests measures the function of both the extensor and flexor muscles. *Ark2C* ^−/−^ animals performed poorly in the test with mean hanging times of less than 15 s (*n* = 8) compared to *wt* mice (*n* = 7), which have mean times close to 60 s, the end-point of the test (Figure 12D). A subpopulation (12.5% of *n* = 16) of *Ark2C* ^+/−^;*BMPRII* ^+/−^ mice had a performance of less than 30 s, suggesting that surviving double heterozygous mice exhibit weakness in forelimbs, albeit less severe than that observed in *Ark2C* ^−/−^ mice. Together the analysis in embryos and adult mice supports the hypothesis that the dorsal limb innervation defect is caused by a reduction in BMP signaling and that Ark2C enhancement plays a compensatory role to maintain high levels of the BMP-activated downstream response.

### Ark2C Enhances Smad-Dependent Signaling Within Motor Neurons

As BMPRII is expressed both in neurons and muscle [54],[55], the above genetic interaction experiment does not reveal whether signaling reduction within MN or the muscle is responsible for the defective axon elongation in the transheterozygote forelimbs. In addition, BMPRII can signal independently of Smads [55]; therefore, it remained unclear whether the phenotype is Smad1/5/8 dependent.

Smad1/5 are expressed broadly in the mouse embryo, including the spinal cord, but Smad8 expression is highly restricted and is nearly absent from the developing nervous system [56]. *Smad8* ^−/−^ mice survive and have been maintained by homozygous breeding [56]. Using this stock of *Smad8* ^−/−^ mice and *Ark2C* ^+/−^ we first generated *Ark2C* ^+/−^ *;Smad8* ^+/−^ double heterozygotes. These were then bred with *Smad8* ^−/−^ mice to generate *Smad8* ^−/−^ (Smad8 null), *Smad8* ^+/−^ (Smad8 het), *Ark2C* ^+/−^;*Smad8* ^+/−^ (double het), and *Ark2C* ^+/−^;*Smad8* ^−/−^ (het hom) compound mutants. Analysis of the above offspring showed that *Ark2C* ^+/−^;*Smad8* ^−/−^ mice are born at lower than expected numbers and exhibit reduced survival prior to weaning (Table S1B). Up to 50% of these (*Ark2C* ^+/−^;*Smad8* ^−/−^) compound mutant adult animals exhibit forelimb movement and posture defects reminiscent of those observed in *Ark2C* ^−/−^, but not *Ark2C* ^+/−^ mice (Figure 4F–H). Assessment of limb weakness on the cage lid test showed that *Ark2C* ^+/−^;*Smad8* ^−/−^ mice exhibit a wide range of performances with 55% of animals failing to reach 30 s (*n* = 11) compared to 7% of littermate controls of mixed genotype (*n* = 15) (Figure 12D). Analysis of the forelimb muscles of poorly performing individuals revealed atrophy with unilateral penetrance in the muscles controlling the digits and wrist (Figure S8C). These are the same muscles that were found to atrophy in a bilateral manner in *Ark2C* ^−/−^ postnatal stage animals (Figure 3A).

Furthermore, MN axon defects in the forelimbs were examined with the HB9-eGFP marker in the above genetic crosses. Thirty out of 52 embryos studied were either *Smad8* ^+/−^, *Smad8* ^−/−^ or *Ark2C* ^+/−^;*Smad8* ^+/−^ and did not exhibit innervation defects in any of their 60 forelimbs. The remaining 22 embryos were *Ark2C* ^+/−^;*Smad8* ^−/−^. Of these 44 forelimbs, 10 forelimbs exhibited a reduction or loss of the most distal innervation (red and blue arrowheads in Figure 12B, and Figure S8A and B) with five showing additional reduction in the innervation of more proximal muscles (green arrowhead, Figure 12B and Figure S8A and B). Quantitation of the results is shown in Figure 12C. The levels of the BMP-Smad signaling response are most likely reduced in *Ark2C* ^+/−^ mice, but they remain above the threshold that is required for efficient advancement of motor axons. However, a reduction of one BMP-effector in these *Ark2C* ^+/−^ animals brings the signaling response below the threshold, thereby reproducing the dorsal forelimb innervation deficit otherwise observed only in *Ark2C* ^−/−^ individuals. The data support the hypothesis that Ark2C enhances BMP-Smad signaling responses in vivo and that this enhancement is required for MN axon elongation particularly in the dorsal forelimb.

Smad8 expression is absent from the muscle and restricted in ventral horns of the brachial spinal cord (Figure 12E), while Ark2C is expressed in the entire spinal cord (Figure 1G–H). As both these factors are involved in controlling dorsal forelimb innervation, their interaction must take place within the spinal cord and specifically in the Smad8 expression domain. We therefore used a *lacZ*-reporter knocked in to the *Smad8* locus [56] to examine the specificity of this expression in detail. This showed that Smad8 is expressed in a few cells of the ventral spinal cord at E12.5–15.5 (Figure 12E and F and unpublished data). Motor pool marker analysis showed that the Smad8-lacZ positive neurons are present within the LMC (FoxP1 domain in Figure 12F). These must be the MN that require Ark2C and Smad8 for their axonal projection. The spinal cord has been shown to express broadly both Smad1 and Smad5 [56]; therefore, the activation of the third effector Smad8 in a subset of MN suggests a special requirement for higher BMP signaling responses in these cells. Collectively, the above genetic experiments show that Ark2C function is to enhance BMP-Smad signaling within MN and that it is essential for the efficient advancement of motor axons in the dorsal forelimb.

## Discussion

Motor axons are amongst the longest in the body and both intrinsic and extrinsic factors have been shown to play a role in their elongation. However, a full understanding of the many signaling pathways that affect the process has not been achieved. In this study we present a collection of evidence supporting that Ark2C and BMP-Smad signaling are involved in motor axon elongation during development.

Ark2C is shown to enhance BMP-Smad signaling responses by gain of function experiments in the chick spinal cord in vivo (Figure 7), and a mechanism whereby Ark2C derepresses the pathway by mediating the degradation of intracellular repressors is described (Figures 8 and 9). Genetic interaction experiments confirm that reduction of Ark2C in vivo along with BMP signaling components recapitulates forelimb innervation deficits otherwise observed only in the complete absence of Ark2C in mice (Figure 12). The proposed role of Ark2C in motor axon projection during development is supported by analysis of *Ark2C* ^−/−^ embryos, showing phenotypes that range from severe reduction of the innervation in the dorsal forelimb to the shortening of presynaptic branches as observed in the phrenic (Figures 4 and 5). Additionally, active BMP-Smad signaling is present in the brachial ventral spinal cord including the LMC (Figure 11) and treatment of cultured NSC-34 or primary MN with BMP-inhibitor diminishes axon elongation (Figure 11).

Although the presence of pSmad1/5/8 is high in DRG during development and recent findings support a role of Smad-dependent BMP signaling in sensory neuron axon regeneration in vivo and in culture [16],[17], the role of this pathway in axon growth in the periphery during development and neuromuscular connectivity remained largely unknown. Moreover, it has been reported that MN do not harbor activated BMP-Smads, and conditional deletion of one of the four BMP type I receptors, BmprIa, revealed that while it has an essential role in limb mesenchyme patterning, it is not required in MN [57]. These experiments do not exclude a role of BMP in motor neurons acting via a different type I receptor. Our finding that phosphorylated Smad1/5/8 are present in motor pool nuclei is based on both immunostaining with a-pSmad1/5/8 (Figure 11H) and PLA, a technique that detects protein interactions (Figure 11G). The latter technique was adapted specifically to detect pSmad1/5/8 and Smad4 complexes that form only upon ligand stimulation [58]. These results confirmed that pSmad1/5/8 are present in motor pool nuclei including the entire LMC and reveal the likely involvement of canonical BMP signaling in MN.

As Ark2C functions to boost signaling intracellularly, its loss is not expected to abolish BMP signaling but instead exposes which cells (MN) require an enhancement of BMP-Smad signaling activity to reach the desired cellular outcome. Despite the expression of Ark2C throughout the spinal cord, the phenotype produced upon its loss indicates that it is required in only a subset of MN (predominantly in the LMC_l_) for neuromuscular connectivity (Figure 4). This suggests that in other MN, BMP signaling is sufficiently high that the desired threshold is reached without intracellular enhancement by Ark2C. The requirement of intracellular boosting of signaling in a subset of LMC neurons is supported by the observed expression of Smad8 in a subpopulation of the LMC in addition to Smad1 and 5, which are widely expressed [51],[56]. When Smad8 expression is lost along with one allele of *Ark2C* in mice, LMC_l_ axons exhibit inefficient projections to their most distal target muscles of the forelimb (Figure 12B and C). Together, these observations propose a hypothesis that the requirement for BMP-Smad signaling in MN axon elongation is broad, but a subset of neurons cannot reach the vital threshold by ligand stimulation alone. Instead, these cells require intracellular enhancement of the pathway, achieved by the presence of the third effector Smad (Smad8) and by a dependency upon Ark2C. The specificity of the requirement for BMP-Smad intracellular enhancement in the dorsal forelimb compartment may be due to fluctuations in BMP ligand availability or in the activation of BMP signaling antagonists.

Measurements of the diameter of the dorsal and ventral nerve trajectories into the forelimb at E11.5 (Figure 4A–D and Figure S2A–F) and 3D projections of innervation to and from the brachial plexus (Figure 4E) do not show evidence of misguidance in Ark2C null mutants. Additionally, there is no evidence of major misspecification of LMC_l_ motor neurons to an LMC_m_ identity to account for the severe reduction of LMC_l_ projections in the absence of Ark2C (Figure 6 and Figure S3). Furthermore, in the absence of Ark2C the dorsal forelimb innervation deficit is not focused only on one muscle group involving a specific LMC_l_ subpopulation. Instead it alters during development and from E13.5 onwards affects predominantly dorsal muscles to a varying degree dependent upon their proximal-distal location (Figure 4F–H). As misrouting and misspecification cannot account for the phenotype in the Ark2C mutants, reduced axon growth is the most plausible explanation. Several lines of evidence including biochemistry (Figures 8 and 9), chick spinal cord functional assays (Figure 7), and genetics (Figure 12) support that Ark2C functions via the BMP-Smad pathway and that this signaling is activated in motor neurons (Figure 11). The above evidence together with the finding that treatment of MN in culture with BMP4 or an inhibitor results in positive or negative effects on axon growth, respectively (Figure 11C–F), suggest that Ark2C-enhanced BMP signaling is involved in axon growth.

BMP ligands are abundant in additional peripheral synaptic targets other than the limbs and have already been shown to activate Smads within sympathetic and trigeminal sensory neurons in a retrograde manner [18],[19],[59]. However, in mammalian MN, this has not been observed. Our findings support the possibility that BMP-Smad stimulation of cells within the LMC also originates from ligands in the periphery. In the case of such retrograde activation, axons projecting to distinct limb compartments would be exposed to variable ligand stimulation. During development several BMP ligands and their antagonists are expressed with dynamic patterns within the limbs as they regulate limb growth and patterning (and Figure S7) [60],[61]. This changing expression pattern may explain the differences in phenotypic severity observed over time (Figure 4) but might also contribute to the initial requirement for Ark2C-mediated signaling enhancement. Prolonged exposure of MN growth cones and synapses to BMP ligands is expected to activate intracellular negative feedback mechanisms [26],[29] that lower the downstream responses, in this case axon extension. Additionally, the peripheral tissue that produces the ligand is expected to activate extracellular antagonists as part of a negative feedback mechanism [23], limiting the amount of BMP available to the growth cones and axons. Under these conditions the presence of Ark2C in MN could derepress BMP signaling and counteract certain negative feedback mechanisms. In this manner the presence of Ark2C in all MN safeguards the levels of BMP signaling responses maintaining the growth of motor axons through an environment with variable and dynamic ligand stimulation. Minor variations in the amount of ligand present in the limb or activation of negative feedback between individual embryos may also explain the variability of the axon extension defects observed in congenic strains of *Ark2C* ^−/−^ embryos or between innervation in different limbs within the same embryo.

In *Drosophila*, retrograde BMP Smad-dependent signaling is required for synaptic growth and plasticity in MN [20]–[22]. However, BMP signaling is not essential for specification, initial axon elongation, or synaptogenesis. The phenotype seen upon reduction of BMP signaling in *Drosophila* MN is associated with the expansion and plasticity of the synapse. This shares aspects of the terminal branch elongation defect observed in the *Ark2C* ^−/−^ phrenic nerve (Figure 5A–C). It is therefore tempting to speculate that BMP signaling is involved in axon and presynaptic lengthening along with synapse expansion in both organisms.

We have revealed that BMP-Smad signaling is involved in MN axon elongation and identified Ark2C as a positive regulator of the pathway participating in this process. Our research is expected to focus future studies on determining whether BMP signaling is involved in MN axon plasticity or degeneration/regeneration and whether it can be used to modulate these events to prevent disease. Future studies could also address how broad the role for BMP signaling is in developmental axon elongation and whether it might underlie disorders associated with neuromuscular connectivity.

## Materials and Methods

### Transgenic Mice and Genotype Analysis

Mice carrying the gene-trap in Ark2C gene were generated from the P9-3f ES cells of the International Gene Trap Consortium (http://www.genetrap.org/; Soriano Lab Gene Trap Database). Ark2C mutant mice were crossed with lines carrying Hb9-eGFP [62] (gift from K.V. Anderson, Memorial Sloan-Kettering, NY), Smad8 LacZ [56] (gift from E.J. Robertson, University of Oxford), and mutated BMP type II receptor [53] (gifts from K. Miyazono, University of Tokyo).

Genotyping was carried out using PCR, as described for Smad8 and BMPRII lines [53],[56], and for Ark2C, with primers: F, 5′-GCTGGGTGCTGTCCTAGAAG-3′; R(*wt*), 5′-CCGGGGTATATGCAATTCTG-3′; R(mut), 5′-ACTGGAAAGACCGCGAAGAG-3′; and the following conditions: 94°C for 5 min, then 39 cycles of 94°C for 30 s, 58°C for 60 s, 72°C for 90 s, and 72°C extension for 5 min. DNA was separated on 1.5% agarose gels resulting in bands at 320 bp (*wt*) and 575 bp (null allele).

### DNA Constructs

Tagged Ark2C constructs were generated by fusing full-length mouse Ark2C or amino acids 68–346 of human Ark2C in frame with GFP (pEGFP-cI; Clontech). The GFP-Ark2C RING deletion mutant was constructed by deleting the last 52 amino acids, which include both zinc fingers, from the human Ark2C. The various tagged Ark2C sequences were subcloned into pTriEx2-hygro (Novagen) at the *Sma*I site (GFP, GFP-mArk2C) or the *Nco*I site (GFP-hArk2C).

The following reporter constructs were used in *in ovo* luciferase assay: 12×-CAGA-Lux, BRE-lux, and pRL-SV40. The expression vectors for mycSnoN, mycSki, FLAG-Ub, mycSmad4, mycSmad6, and mycSmad7 are described previously [31],[32]. The expression vectors pCDEF-FLAG-Ark2N and pCDEF-FLAG-Ark2C are gifts from K. Miyazono.

### Cell Lines and Transient Transfection

Human embryonic kidney cells (293T), NSC-34, and Arkadia-null mouse embryonic fibroblasts were cultured in Dulbecco's Modified Eagle's Medium supplemented with 10% fetal bovine serum, L-glutamine, and penicillin/streptomycin at 37°C in a 5% CO_2_ atmosphere. Transient transfections were performed using Lipofectamine 2000 according to the manufacturer's instructions.

### Quantitative and Semiquantitative RT-PCR Analysis

Total RNA was extracted and purified using the RNeasy Mini kit with on-column DNaseI treatment (Qiagen). Reverse transcription reactions were carried out using the Superscript III First Strand Synthesis System for RT-PCR (Invitrogen). Primers used are shown in Table S2, and reactions were normalized against GAPDH or YWHAZ.

### X-Gal Staining

Whole-mount tissues and 100 µm vibratome sections were fixed in X-gal fix, washed in X-gal rinse, and stained as described [34]. Post X-gal stain immunocytochemistry was carried out using Ultra Sensitive ABC Peroxidase Kit and Metal Enhanced DAB Substrate (Thermo Scientific).

### In Ovo Electroporation and Luciferase Assays

Constructs were electroporated into the neural tube of HH stage 11–12 chick embryos. The renilla luciferase was used at 0.1 µg/µl, luciferase reporter and test DNA at 1 µg/µl each. When exogenous ligand was added to the DNA mix, it made up 1/10 of the test DNA. The spinal cord was harvested after 22–24 h and luciferase activity measured using the Dual Luciferase Reporter Assay (Promega) [63].

The firefly/renilla luciferase ratio for each chick spinal cord or well of cells was calculated; graphs show mean for each population and the standard error of the mean. Two-tailed homoscedastic Student's *t* tests were used to calculate the probability of different populations being identical.

### In Situ Hybridization and Immunofluorescence

Whole-mount *in situ* hybridization of Ark2C was carried out as described [40],[64] using a 3′ UTR probe described in http://mouse.brain-map.org/brain/Gm96.html. BMP probes were used as described [65]–[67].

Whole-mount antibody staining of mouse forelimb motor axons was performed as described [40] using a-GFP (1∶500, Invitrogen, A11122) and a-myosin-32 (1∶500, Sigma, M4276). Dissections were carried out at different times of day to obtain intermediate stage embryos. GFP-labeled motor axons were imaged using a Leica SP5 confocal microscope, stacks were taken through the entire extent of the forelimb (approximately 500 µm depth), and images stitched using Leica software. The 3D projections were created using ImageJ software and movies using VideoMach (under the advice of Dirk Dorman microscopy laboratory, MRC CSC). Measurements of both major dorsal and ventral axon bundles originating from the brachial plexus and the spinal nerves were made at E11.5 using ImageJ. Width of spinal nerves was measured at the point of amalgamation with another nerve (Figure S2A and B), and the area of the spinal nerves and brachial plexus was measured as shown in Figure S2A. Length measurements of nerves within the limb were made following the approximate trajectory of axons from a line drawn across the brachial plexus made using landmarks to ensure consistent positioning. Width of the axon bundle was measured at three points along its length, avoiding regions proximal to the plexus, branch points, or growth cones where the bundle was wider. Volumetric measurements of the brachial plexus were obtained using Imaris software.

Whole-mount staining of diaphragms was carried out as described [68] using a-neurofilament (1∶1,000, Chemicon, AB1981), a-synaptophysin (1∶100, Zymed/Invitrogen, 18-0130), and Alexa Fluro 488 α-Bungarotoxin (1∶250, Invitrogen, B13422). Image stacks were taken at a ventral location within the diaphragm muscle to prevent secondary nerve branches complicating measurements. Stacks were flattened and the number of synapses present and length of phrenic nerve within the image were measured. Fifteen synapses towards the outer edge of the endplate were chosen at random and their terminal branches were traced using Neuron J (1 *wt* and 2 *Akd2C* ^−/−^ diaphragms at E17.5, 2 *wt* and 2 *Akd2C* ^−/−^ diaphragms at P17).

Antibody staining was performed on 14 µm cryosections using guinea pig a-FoxP1 (1∶16,000), rabbit a-FoxP1 (1∶32,000), guinea pig a-Hb9 (1∶16,000), rabbit a-Isl1 (1∶4,000, all gifts from T. Jessell), rabbit a-Pea3 (1∶5,000, gift from S. Arber), a-pSmad1/5/8 (1∶1,000, Cell Signaling, 9511), and rabbit a-β-galactosidase (1 µg/ml, MP Biomedicals, 08559762). Multiple sections from 13 embryos of each genotype were analyzed (in total 2,042 *wt* and 2,452 *Akd2C* ^−/−^ FoxP1 expressing cells were counted, average of 51.16 and 49.32 cells per section), and the mean percentage of Foxp1 positive cells expressing a second gene was calculated.

### Footprint Test

The forepaws of the mouse were painted with black food colouring and the mouse walked on paper for 30 cm. Animals with abnormal trails were tested at least twice. The resulting pawprints were then analysed using some of the parameters described in [69].

### Hypoxia Test

Resting adult mice were warmed at 39°C for 15 min and 100 µl of blood were taken from the tip of the tail. Blood plasma was separated by centrifugation for 15 min at 4,000 rpm. Lactate levels in the plasma were measured using a Lactate Colorimetric Assay Kit (Abcam).

### Strength and Grip Neurological Test

A mouse was placed on the wire cage-lid, and the lid was inverted and held over the cage at about 25 cm. The time before the mouse fell was measured with a cut-off time of 60 s [70]. Two tests were carried out 20 min apart; this was repeated at weekly intervals at least three times. To check for increasing phenotype severity as the animals aged, the protocol was carried out weekly for 4 months.

### Immunoprecipitation (IP) Assays/Immunoblotting (IB)

For the IP assays, cells were treated with 2 µM dorsomorphin (DM; a selective inhibitor of BMP type I receptors; Merck) to achieve BMP inhibition, 25 ng/ml BMP4 for stimulation (R&D), 20 µM SB431542 (S4317) to inhibit TGF-β signaling, or 10 ng/ml Activin A (A4941, Sigma) for stimulation. All treatments were done in serum-free medium for 1 h in the presence of 20–50 µM of the proteasome inhibitor MG132 (Sigma). After the treatments, cells were lysed in lysis buffer (20 mM Tris−HCl, pH 7.5, 150 mM NaCl, 10% glycerol, and 1% Triton X-100) supplemented with protease and phosphatase inhibitors (Roche), and 50 µM MG132. Cell lysates were incubated with an a-GFP antibody (Roche, 11814460001) and dynabeads (Invitrogen) for 4 h at 4°C. After extensive washes with lysis buffer, the proteins were eluted with sample buffer and analyzed by IB.

For the protein kinetics, cells were treated with 2 µM DM or 25 ng/ml BMP4 in serum-free medium or left untreated in medium containing 10% FBS. The cells were lysed in the above lysis buffer and the samples were analyzed by IB. Quantitation was performed with the ImageJ software.

The antibodies used in the IB are a-pSmad1/5/8 (1∶500), a-Smad1/5/8 (1∶500, Santa Cruz, sc-6031-R), a-pSmad2 (1∶500, Cell Signaling, 3101), a-Smad6/7 (1∶1,000, Santa Cruz, sc-7004), a-Ski (1∶3,000, Millipore), a-SnoN (1∶5,000, Santa Cruz, sc-9141), a-PCNA (1∶10,000, Chemicon, MAB424), a-myc (1∶500, Sigma, M5546), a-FLAG (1∶1,000, Sigma, F1804), and a-GFP (1∶1,000, Invitrogen). Experiments were performed two to three times.

### Detection of Ubiquitinated Proteins

Cells were treated with 20 µM MG132 for 4 h, harvested in PBS, and lysed in 1% SDS. Lysates were boiled for 5 min at 95°C and diluted with dissociation buffer (1% TritonX-100, 0.5% sodium deoxycholate, 120 mM NaCl, 50 mM Hepes pH 7.2, supplemented with protease inhibitors and 50 µM MG132) to a final SDS concentration of 0.1%. The cleared lysates were incubated with a-myc antibody (Santa Cruz, sc-789) and dynabeads (Invitrogen) for 4 h at 4°C. Bound proteins were washed extensively with dissociation buffer and eluted in sample buffer. The bound proteins as well as the total lysates (TLs) were analyzed by IB using a-FLAG and a-myc antibodies (Sigma). Experiments were performed twice and representative images are shown in Figure 7C–F.

### *In Situ* Proximity ligation (PLA)

*In situ* proximity ligation was performed using the Duolink or Duolink II kit according to the manufacturer's instructions (Olink Biosciences) and as described [58]. E13.5 mouse embryos were dissected, fixed in 4% PFA for 2 h on ice, and embedded in 4% agar. We prepared 100 µm vibratome sections from the brachial level. The cells and the vibratome sections were permeabilized with PBS-TritonX-100 0.5% and then incubated with blocking solution (Olink Biosciences). The samples were incubated with the primary antibodies [a-pSmad1/5/8 (1∶100), a-Ski (1∶100), a-FLAG (1∶100), and a-Smad4 (1∶100, Santa Cruz, sc-7966)] for 1 h at 37°C. The secondary probes used were Duolink anti-Rabbit PLUS (90302) and Duolink anti-Mouse MINUS (90301) or Duolink II PLA probe anti-Rabbit PLUS (92002) and Duolink II PLA probe anti-Mouse MINUS (92004). The detection reagents used were Duolink detection kit 563 (90104) or Duolink II Orange (92007), and images were acquired using a Leica SP5 confocal microscope. The quantification of PLA signal was performed using the ImageTool software (Olink Biosciences).

### Neurite Outgrowth Assay and Immunocytochemistry in NSC-34 Cells and Primary MN

NSC-34 cells were differentiated as described [71] on coverslips coated with laminin (Sigma) and lysine (Sigma). The cells were cultured in DMEM supplemented with 1% FBS, 1% FBS, and 2 µM dorsomorphin or 1% FBS and 25 ng/ml BMP4 for 48 or 72 h. The concentration of dorsomorphin was chosen after titration (0.5–5 µM) to avoid toxicity. The cells were stained with a-Neurofilament (1∶1,000), and images were acquired using a Nikon Eclipse E1000M microscope at 4× and 20× magnification. Neurite outgrowth was measured as the percentage of cells that have axons grown more than 65 µm from the center of the cell body (Figure S5D) to the total number of cells for each condition. Axons were measured from at least five images of six different experiments (400–425 cells in total). Two-tailed homoscedastic Student's *t* tests were used to calculate the probability of different populations being identical.

Primary MN were isolated from Hb9-eGFP mouse embryos at E13.5 and purified by density centrifugation according to a modified protocol by Mazarakis and Schiavo based on published protocols [72],[73]. The cells were plated on Permanox chamber slides (LabTek) coated with poly-L-ornithine and laminin (Sigma). MN were maintained in Neurobasal medium (Invitrogen) supplemented with GDNF (10 µg/ml), CTNF (10 µg/ml), and BDNF (10 µg/ml) (Alomone Labs). After an overnight incubation the cells were treated for 72 h with 2 µM dorsomorphin or 25 ng/ml BMP4 and then fixed with 4% PFA and stained with a-GFP (Roche). GFP-labeled motor axons were imaged using a Leica SP5 confocal microscope, and their length was measured using the NeuronJ plugin of ImageJ. Two-tailed homoscedastic Student's *t* tests were used to calculate the probability of different populations being identical.

### Accession Numbers

GenBank (http://www.ncbi.nih.gov/Genbank/) accession numbers for the genes discussed in this paper are Bmpr1a (NM_009758).

## Supporting Information

Figure S1 Early expression of the novel Arkadia-like gene, Ark2C. (A) Ideogram showing significant alignments of the mouse *Arkadia* cDNA sequence (blue arrow) in the mouse genome using BLAST. Two alignments on chromosome 3 (green arrows) are Arkadia-like pseudogenes. Ark2 (red arrow) is located on chromosome 18. (B) Whole-mount in situ hybridization with Ark2C anti-sense and sense probes as indicated, in the early embryo. HF, headfold; E7.5 embryo is showed from a ventral view point; scale bars = 100 µm (E6.5, E7.5) and 500 µm (E9.5).
(TIF)

Figure S2 Reduction in motor neuron axon growth is observed in *Ark2C* ^−/−^ embryos. (A) Confocal image of whole-mount IF with anti-GFP showing forelimb nerves measured in E11.5 HB9-eGFP transgenic embryos. Proximal limb to the left; a, axillary nerve; r, radial nerve; m, median nerve; u, ulnar nerve; t, thoracodorsal; C5–8 and T1, spinal nerves from appropriate segments. (B) Schematic representation of forelimb motor innervation at E11.5. Blue lines indicate measurement of nerve length from the end of the brachial plexus (dashed red line), orange arrows indicate measurement of nerve width (mean of three measurements), orange lines indicate point of spinal nerve measurements, and blue dashed lines delineate areas measured. (C–D) Quantification of length and width of the axillary, median, and thoracodorsal nerves at E11.5; error bars represent ±SD; ** *p*<0.01; N, number of forelimbs (C–F). (E–F) Quantification of spinal nerve width and area and brachial plexus area; error bars represent ±SD; all *p* values are not significant. (G) Confocal images of whole-mount IF showing developing forelimb extensor muscle and innervation in HB9-eGFP transgenic embryos at E13.5; green, Hb9-eGFP expressing MN; red, myosin-32 expressing muscle; genotype of embryos as indicated. Numbered arrowheads indicate radial nerve partitions as shown in the diagram on Figure 4F; proximal limb to the left; scale bars = 250 µm.
(TIF)

Figure S3 Motor neuron specification is normal in the absence of Ark2C expression. (A) Diagram summarizing molecular marker expression of motor pools innervating the forelimb and diaphragm. LMC, lateral motor column; MMC, medial motor column [76]. (B) Confocal images from brachial spinal cord cryostat sections stained with IF motor pool marker Pea3 and FoxP1 at E13.5. Scale bars = 50 µm. (C) Histogram showing percentages of the number of FoxP1 expressing nuclei in the LMC that also express Pea3; N, number of cells.
(TIF)

Figure S4 Ark2C enhances the phosphorylation of Smad1/5/8 and degradation of negative regulators of the pathway during treatment with BMP4. (A–B) IB showing pSmad1/5/8 in 293T cells transfected with FLAG-Ark2N or FLAG-Ark2C and treated as indicated. Protein levels of pSmad1/5/8 were quantified, normalized to PCNA, and the relative protein levels are shown in arbitrary units in the histograph. (C–F) Repeats of the IB shown in Figure 8: Smad6/7 (C) and Ski (E). The histograph in (F) is the average of values from the two IBs presented in (E). The trend of protein levels is similar to that shown in Figure 8C–F. Untr, untreated; arrows indicate specific bands. (G) IB of pSmad1/5/8 steady-state levels after overnight culture with 10% FBS containing BMP. (H) Histograph of the IB in (G). Protein levels were quantified normalized to PCNA, and the relative protein levels are shown in arbitrary units in the histographs.
(TIF)

Figure S5 Ark2C interacts with components of the BMP pathway but not with pSmad2/3. (A) IP in 293T-GArk2C cells after 1 h treatment with SB 431542 (SB), an inhibitor of the TGF-β/Activin pathway, or Activin showing no interaction of GArk2C with pSmad2. TL, total lysate; IB, immunoblot; *, nonspecific band. (B) Confocal images taken from Proximity Ligation Assay (PLA) performed in HEK293T transfected with FLAG-Ark2C and treated for 1 h as indicated showing interaction of Ark2C with endogenous pS1/5/8 or Ski. The omission of the primary antibodies or the use of a single antibody served as negative controls. Red spots, PLA signal; blue, DAPI-nucleus; scale bars = 10 µm.
(TIF)

Figure S6 NSC-34 express Ark2C and MN marker. (A) QPCR showing expression of exon7–8 of *Ark2C* (including RING domain) in NSC-34 cells in 10% and 1% FBS. Expression of *Ark2C* in *wt* and *Ark2C* ^−/−^ in early embryonic brain was used as controls. Error bars represent ±SD. (B) IF showing Isl1 expression (red) in NSC-34 but not in mouse embryonic fibroblasts (MEF). Scale bars = 25 µm. (C) Representative images from IF showing neurofilament in NSC-34 cells after 48 h treatment with 1% FBS+dorsomorphin (Inhibitor) or 1% FBS+BMP4. Untreated cells were maintained in 1% FBS. Cells with long axons (>65 µm from the centre of the cell body) were counted (as shown in D) and percentage to total is shown in a histograph. Scale bars = 200 µm; N, number of cells; error bars represent ±SD between experiments and reflect counts in different slides.
(TIF)

Figure S7 BMP ligands are expressed in the periphery where innervation defects are observed in the absence of Ark2C. (A) Semiquantitative RT-PCR showing expression of BMP2, 4, 6, and 7 in diaphragms from four individual E15.5 embryos. At this developmental stage the phrenic nerve has entered the muscle and is forming terminal branches. (B) *In situ* hybridization showing expression of BMP2, 4, and 7 in the forelimb at E11.5 and E13.5 (n of four litters at each age, 5–10 embryos per litter). Red arrowhead, dorsal BMP expression; blue arrowhead, ventral BMP expression; D, dorsal; V, ventral; Pr, proximal; Di, distal.
(TIF)

Figure S8 Innervation defects are observed in *Ark2C* ^+/−^ *Smad8* ^−/−^ dorsal forelimb. (A) Schematic representation of the major phenotypes seen in *Ark2C* ^+/−^ *Smad8* ^−/−^ embryos at E13.5. Using the same key for the partition of the radial nerve and the corresponding muscle groups that they innervate as shown in Figure 4F: Blue (1) and red (2) arrows show branches innervating muscles that include EDC and EDQ; green arrow (3) shows branches innervating ECRB and ECRL; orange arrow (4), innervation of more proximal regions of the dorsal forelimb. Dotted line indicates reduction of the radial nerve along with its partitions. (B) Confocal image stacks containing mostly the radial nerve from the images in Figure 12B. Arrows as described in (A); proximal limb to the left; scale bars = 250 µm. (C) Extensor muscles from *Ark2C* ^+/−^ *Smad8* ^−/−^ adult mice. ECRL, extensor carpi radialis longus; ECRB, extensor carpi radialis brevis; EDC, extensor digitorum communis; EDQ, extensor digiti quinti; R, right limb; L, left limb; scale bars = 1 mm. No EDC or EDQ was recovered from this *Ark2C* ^+/−^ *Smad8* ^−/−^ mouse.
(TIF)

Table S1 Survival rates of offspring from genetic interactions with Ark2C. (A) *Ark2C* ^+/−^:*BmprII* ^+/−^×*Ark2C* ^+/−^ cross and (B) *Ark2C^+/−^:Smad8^+/−^*×*Ark2C^+/−^:Smad8^+/−^* cross. Tables show expected percentage of total births (according to Mendelian ratios) for each genotype, the actual percentage of total births observed (all animals alive less than 24 h after birth), and the percentage of those animals born that reach weaning age (21 d). *p*<0.001 for numbers born in *Ark2C/BmprII* cross including and excluding *Ark2C* ^−/−^ pups (χ^2^ test, 5 and 3 degrees of freedom, respectively), and *p*<0.01 for numbers weaned in *Ark2C/Smad8* cross including and excluding *Ark2C* ^−/−^ pups (χ^2^ test, 8 and 5 degrees of freedom, respectively).
(TIF)

Table S2 Quantitative PCR primers. Sequences of primers used in all quantitative and semiquantitative RT-PCR.
(TIF)

Movie S1 Wild-type forelimb motor innervation. Movie generated from confocal images of whole-mount IF with anti-GFP showing forelimb innervation at E13.5 in a *wt* HB9-eGFP transgenic embryo projected in 3D rotating around the proximal-distal axis.
(MPEG)

Movie S2 Ark2C-null forelimb motor innervation. Movie generated from confocal images of whole-mount IF with anti-GFP showing forelimb innervation at E13.5 in an *Ark2C* ^−/−^ HB9-eGFP transgenic embryo projected in 3D rotating around the proximal–distal axis.
(MP4)

## Abbreviations

BMP: bone morphogenetic protein
BMPRI/BMPRII: bone morphogenetic protein receptor I/II
E[number]: embryonic day[number]
GFP: green fluorescent protein
MN: motor neuron(s)
PFA: paraformaldehyde
TGF-β: transforming growth factor β
