# Supplementary figures and images for: Rnf165/Ark2C Enhances BMP-Smad Signaling to Mediate Motor Axon Extension
Source: PLoS Biol. 2013 Apr 16;11(4):e1001538. doi: 10.1371/journal.pbio.1001538 (PMC3627648; doi:10.1371/journal.pbio.1001538)

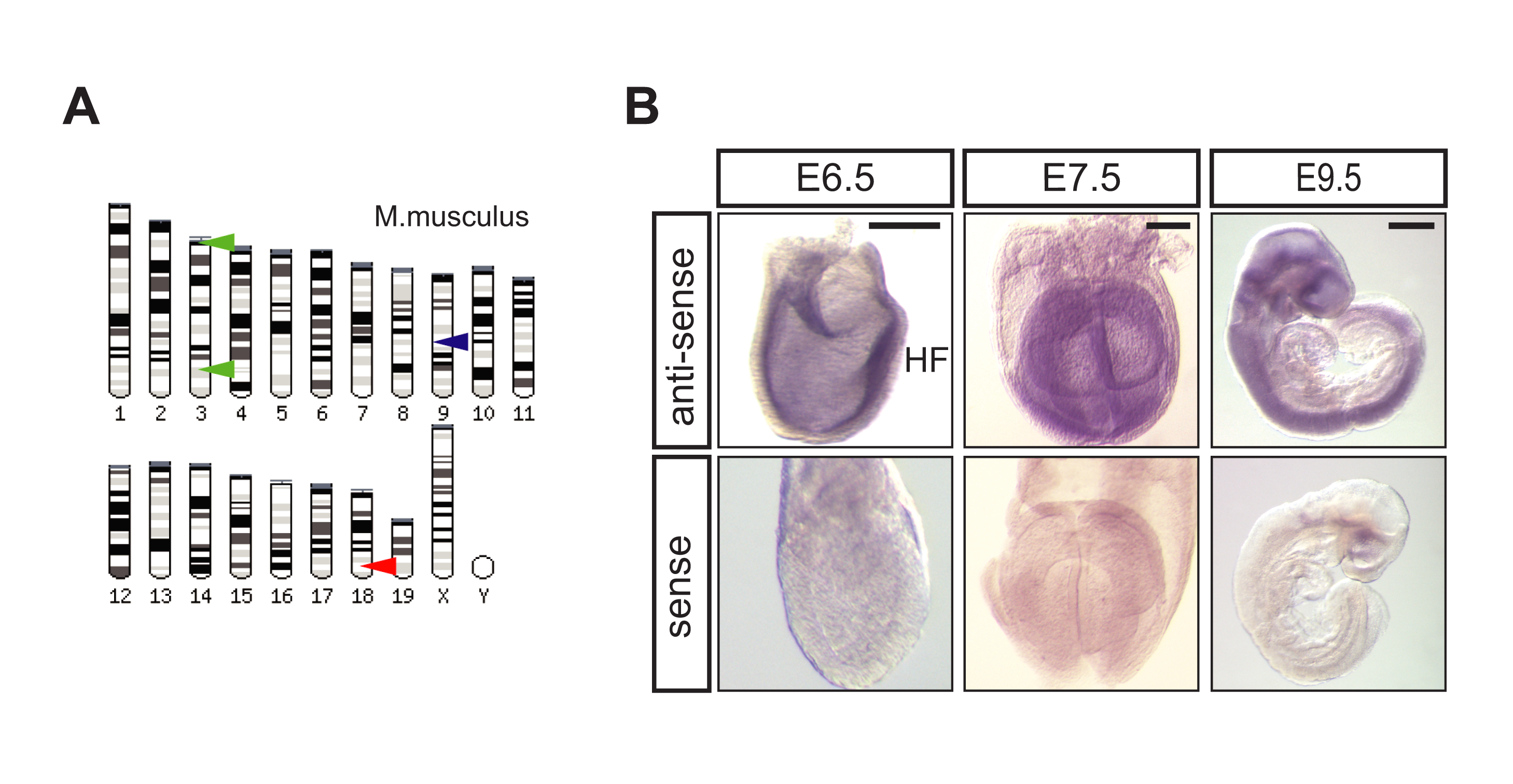

Supplement: Figure S1 — Early expression of the novel Arkadia-like gene, Ark2C. (A) Ideogram showing significant alignments of the mouse Arkadia cDNA sequence (blue arrow) in the mouse genome using BLAST. Two alignments on chromosome 3 (green arrows) are Arkadia-like pseudogenes. Ark2 (red arrow) is located on chromosome 18. (B) Whole-mount in situ hybridization with Ark2C anti-sense and sense probes as indicated, in the early embryo. HF, headfold; E7.5 embryo is showed from a ventral view point; scale bars = 100 µm (E6.5, E7.5) and 500 µm (E9.5). (TIF) [file pbio.1001538.s001.tif]

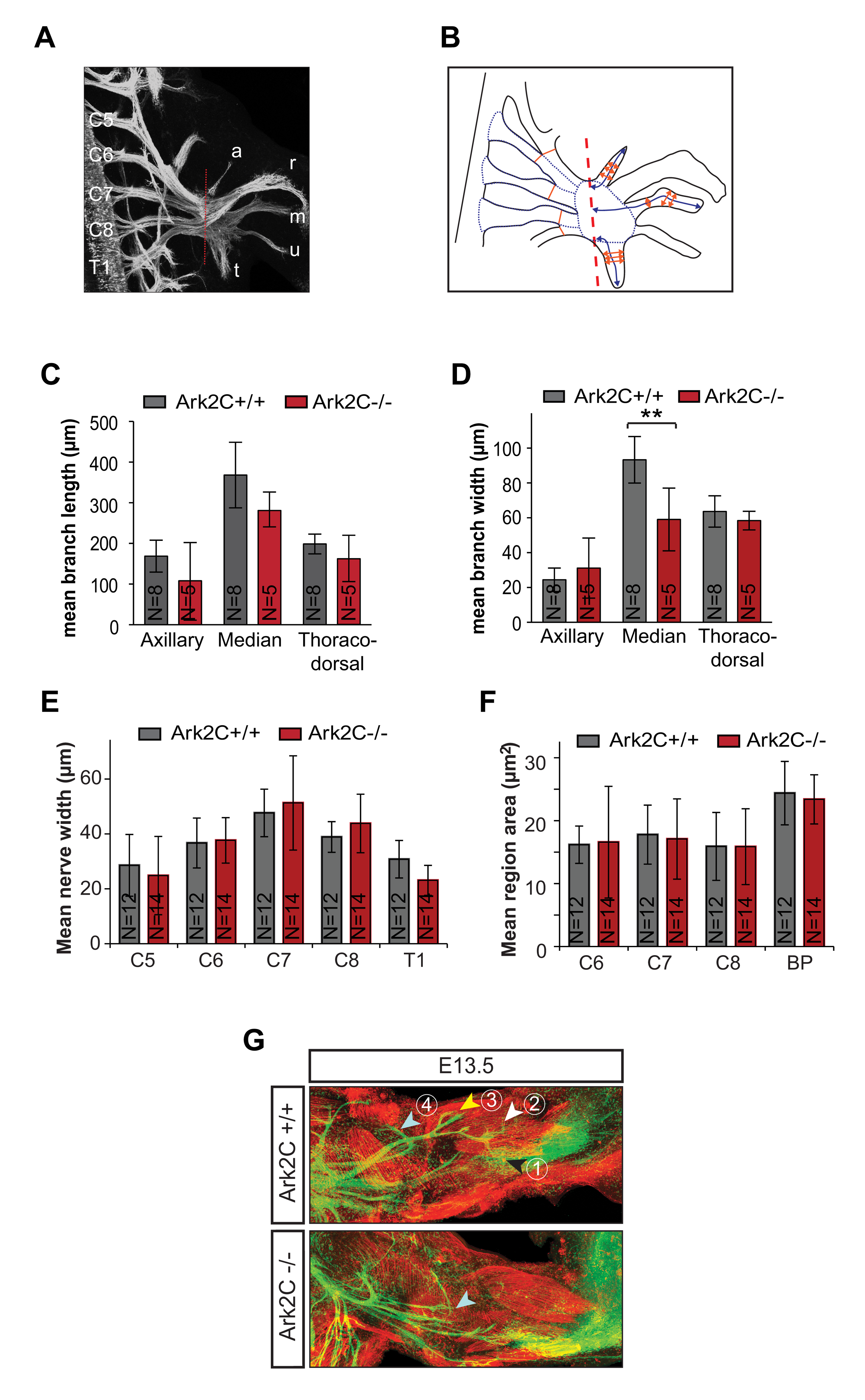

Supplement: Figure S2 — Reduction in motor neuron axon growth is observed in Ark2C −/− embryos. (A) Confocal image of whole-mount IF with anti-GFP showing forelimb nerves measured in E11.5 HB9-eGFP transgenic embryos. Proximal limb to the left; a, axillary nerve; r, radial nerve; m, median nerve; u, ulnar nerve; t, thoracodorsal; C5–8 and T1, spinal nerves from appropriate segments. (B) Schematic representation of forelimb motor innervation at E11.5. Blue lines indicate measurement of nerve length from the end of the brachial plexus (dashed red line), orange arrows indicate measurement of nerve width (mean of three measurements), orange lines indicate point of spinal nerve measurements, and blue dashed lines delineate areas measured. (C–D) Quantification of length and width of the axillary, median, and thoracodorsal nerves at E11.5; error bars represent ±SD; ** p<0.01; N, number of forelimbs (C–F). (E–F) Quantification of spinal nerve width and area and brachial plexus area; error bars represent ±SD; all p values are not significant. (G) Confocal images of whole-mount IF showing developing forelimb extensor muscle and innervation in HB9-eGFP transgenic embryos at E13.5; green, Hb9-eGFP expressing MN; red, myosin-32 expressing muscle; genotype of embryos as indicated. Numbered arrowheads indicate radial nerve partitions as shown in the diagram on Figure 4F; proximal limb to the left; scale bars = 250 µm. (TIF) [file pbio.1001538.s002.tif]

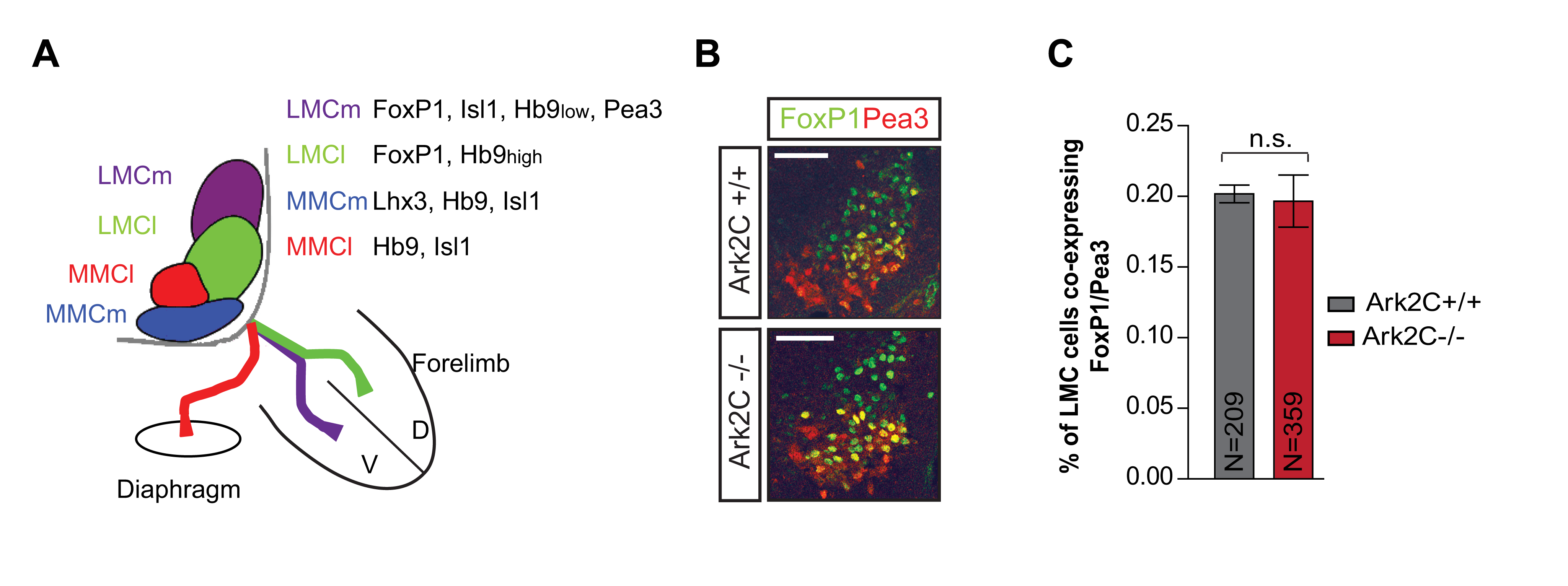

Supplement: Figure S3 — Motor neuron specification is normal in the absence of Ark2C expression. (A) Diagram summarizing molecular marker expression of motor pools innervating the forelimb and diaphragm. LMC, lateral motor column; MMC, medial motor column [76]. (B) Confocal images from brachial spinal cord cryostat sections stained with IF motor pool marker Pea3 and FoxP1 at E13.5. Scale bars = 50 µm. (C) Histogram showing percentages of the number of FoxP1 expressing nuclei in the LMC that also express Pea3; N, number of cells. (TIF) [file pbio.1001538.s003.tif]

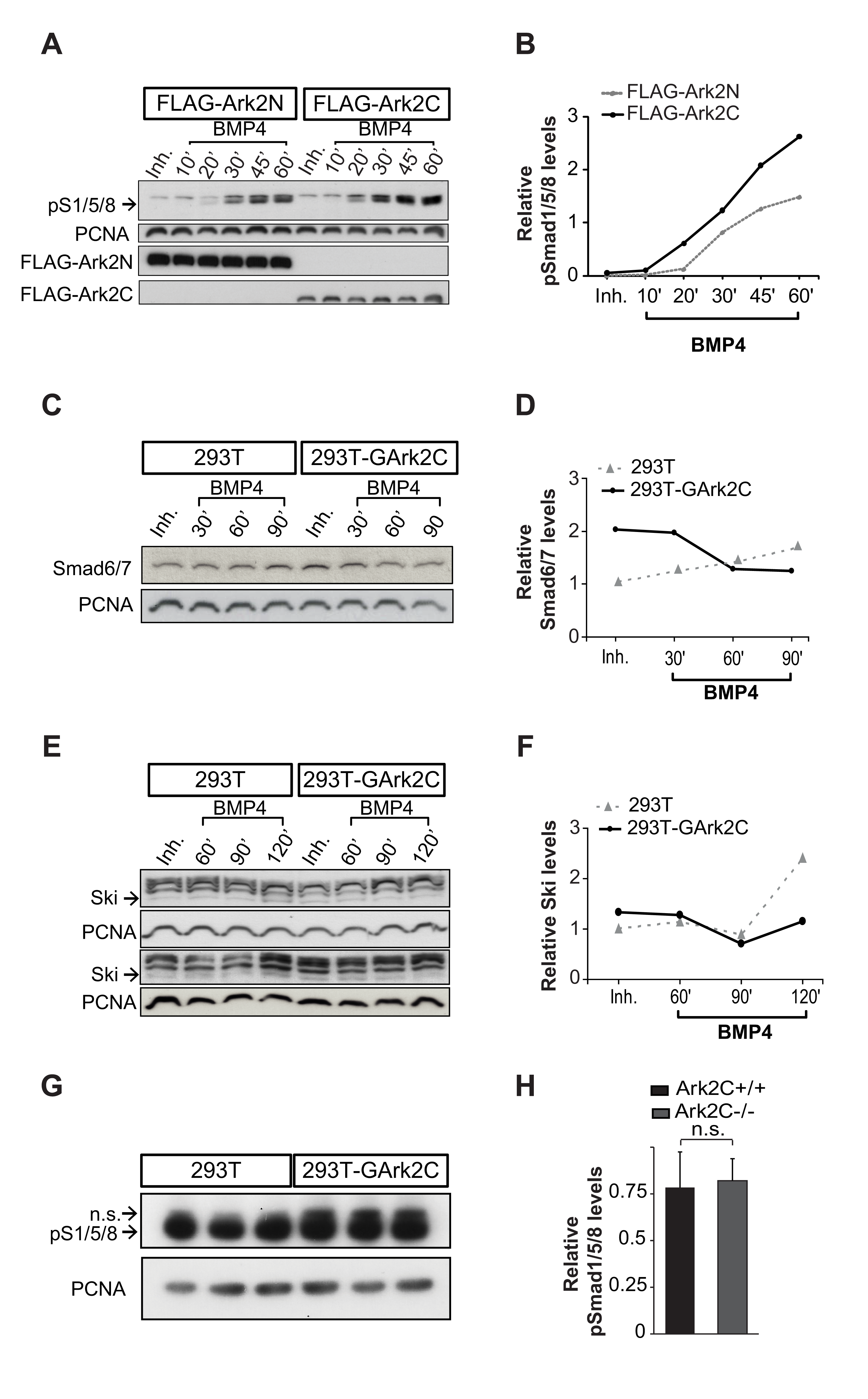

Supplement: Figure S4 — Ark2C enhances the phosphorylation of Smad1/5/8 and degradation of negative regulators of the pathway during treatment with BMP4. (A–B) IB showing pSmad1/5/8 in 293T cells transfected with FLAG-Ark2N or FLAG-Ark2C and treated as indicated. Protein levels of pSmad1/5/8 were quantified, normalized to PCNA, and the relative protein levels are shown in arbitrary units in the histograph. (C–F) Repeats of the IB shown in Figure 8: Smad6/7 (C) and Ski (E). The histograph in (F) is the average of values from the two IBs presented in (E). The trend of protein levels is similar to that shown in Figure 8C–F. Untr, untreated; arrows indicate specific bands. (G) IB of pSmad1/5/8 steady-state levels after overnight culture with 10% FBS containing BMP. (H) Histograph of the IB in (G). Protein levels were quantified normalized to PCNA, and the relative protein levels are shown in arbitrary units in the histographs. (TIF) [file pbio.1001538.s004.tif]

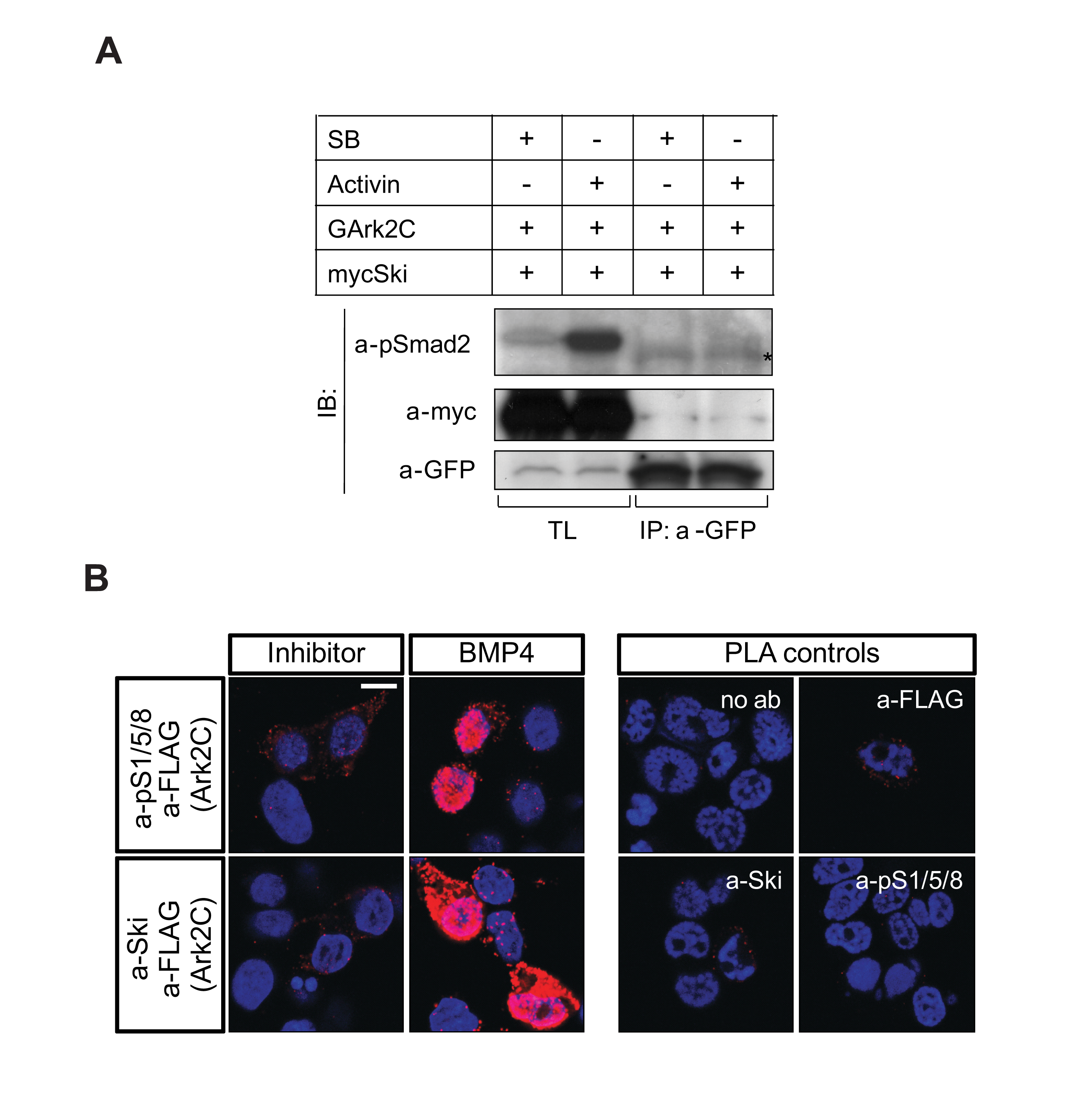

Supplement: Figure S5 — Ark2C interacts with components of the BMP pathway but not with pSmad2/3. (A) IP in 293T-GArk2C cells after 1 h treatment with SB 431542 (SB), an inhibitor of the TGF-β/Activin pathway, or Activin showing no interaction of GArk2C with pSmad2. TL, total lysate; IB, immunoblot; *, nonspecific band. (B) Confocal images taken from Proximity Ligation Assay (PLA) performed in HEK293T transfected with FLAG-Ark2C and treated for 1 h as indicated showing interaction of Ark2C with endogenous pS1/5/8 or Ski. The omission of the primary antibodies or the use of a single antibody served as negative controls. Red spots, PLA signal; blue, DAPI-nucleus; scale bars = 10 µm. (TIF) [file pbio.1001538.s005.tif]

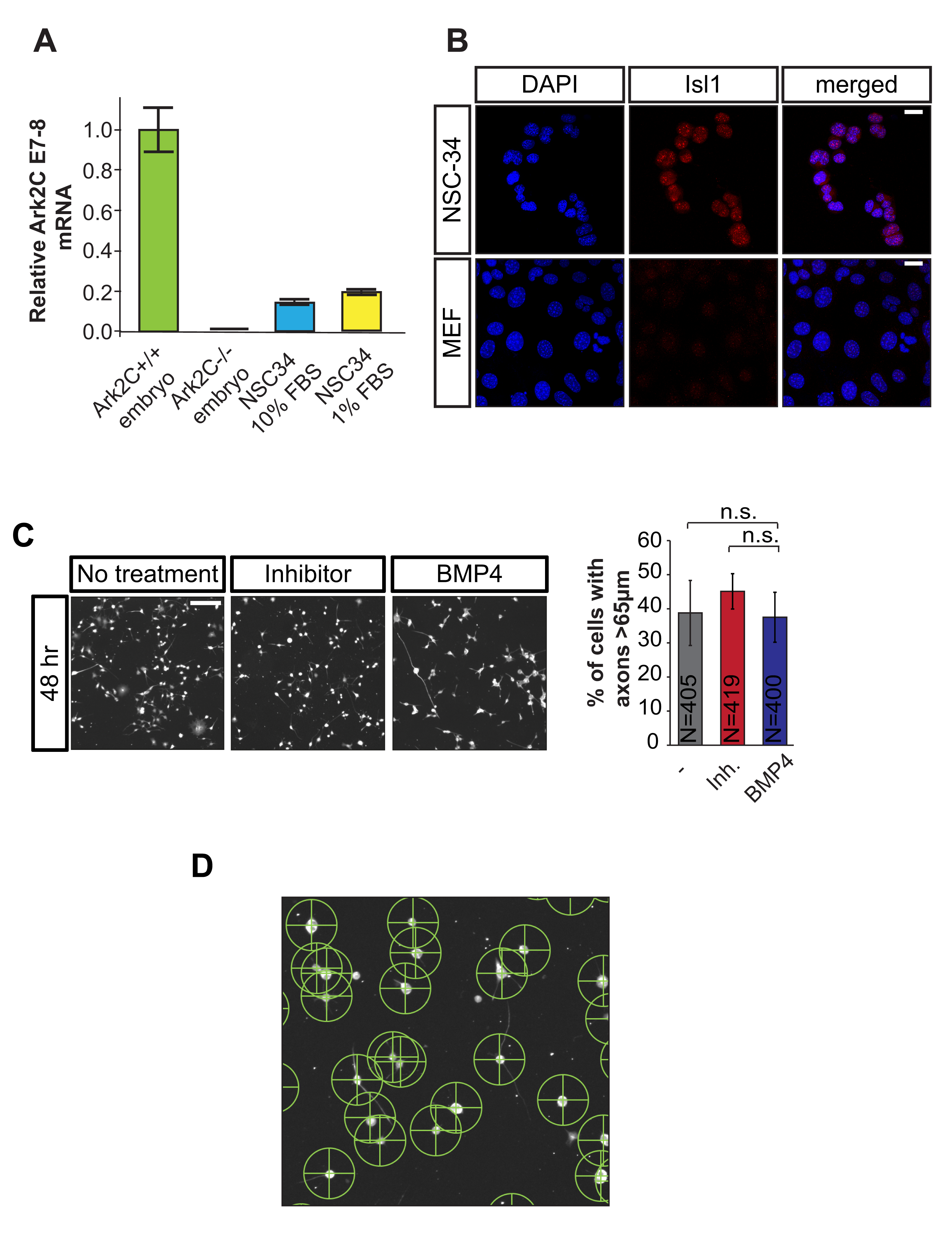

Supplement: Figure S6 — NSC-34 express Ark2C and MN marker. (A) QPCR showing expression of exon7–8 of Ark2C (including RING domain) in NSC-34 cells in 10% and 1% FBS. Expression of Ark2C in wt and Ark2C −/− in early embryonic brain was used as controls. Error bars represent ±SD. (B) IF showing Isl1 expression (red) in NSC-34 but not in mouse embryonic fibroblasts (MEF). Scale bars = 25 µm. (C) Representative images from IF showing neurofilament in NSC-34 cells after 48 h treatment with 1% FBS+dorsomorphin (Inhibitor) or 1% FBS+BMP4. Untreated cells were maintained in 1% FBS. Cells with long axons (>65 µm from the centre of the cell body) were counted (as shown in D) and percentage to total is shown in a histograph. Scale bars = 200 µm; N, number of cells; error bars represent ±SD between experiments and reflect counts in different slides. (TIF) [file pbio.1001538.s006.tif]

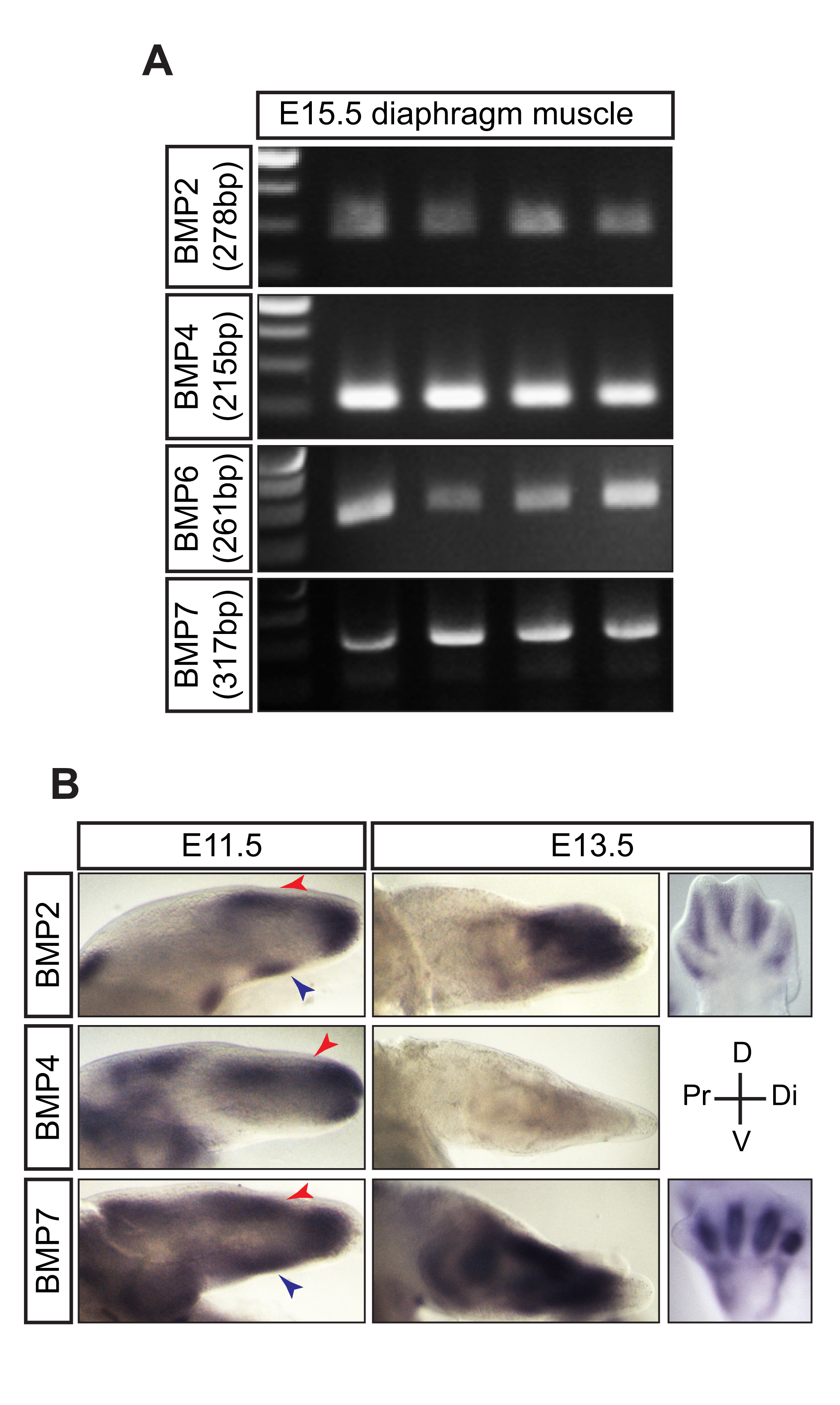

Supplement: Figure S7 — BMP ligands are expressed in the periphery where innervation defects are observed in the absence of Ark2C. (A) Semiquantitative RT-PCR showing expression of BMP2, 4, 6, and 7 in diaphragms from four individual E15.5 embryos. At this developmental stage the phrenic nerve has entered the muscle and is forming terminal branches. (B) In situ hybridization showing expression of BMP2, 4, and 7 in the forelimb at E11.5 and E13.5 (n of four litters at each age, 5–10 embryos per litter). Red arrowhead, dorsal BMP expression; blue arrowhead, ventral BMP expression; D, dorsal; V, ventral; Pr, proximal; Di, distal. (TIF) [file pbio.1001538.s007.tif]

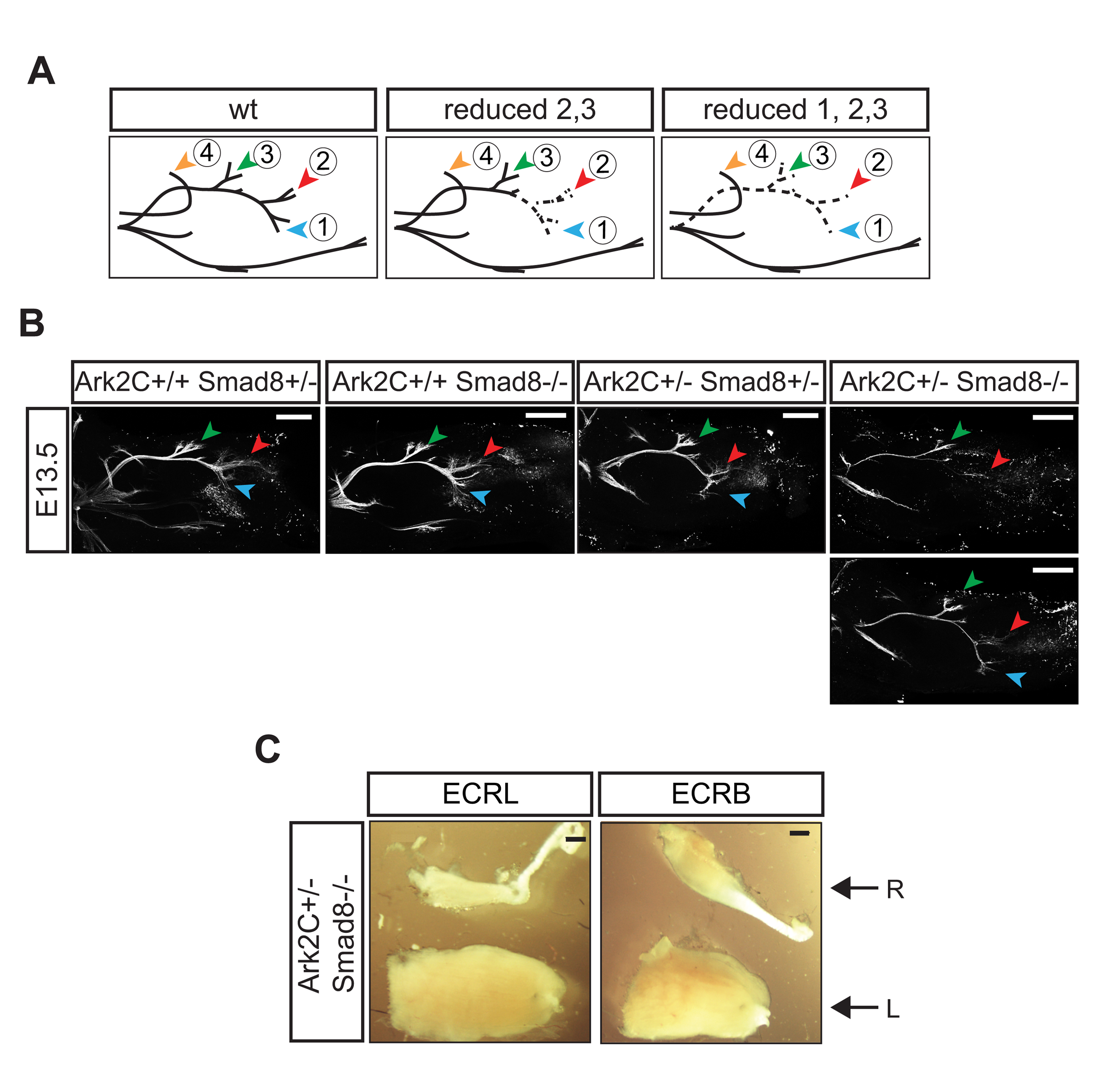

Supplement: Figure S8 — Innervation defects are observed in Ark2C +/− Smad8 −/− dorsal forelimb. (A) Schematic representation of the major phenotypes seen in Ark2C +/− Smad8 −/− embryos at E13.5. Using the same key for the partition of the radial nerve and the corresponding muscle groups that they innervate as shown in Figure 4F: Blue (1) and red (2) arrows show branches innervating muscles that include EDC and EDQ; green arrow (3) shows branches innervating ECRB and ECRL; orange arrow (4), innervation of more proximal regions of the dorsal forelimb. Dotted line indicates reduction of the radial nerve along with its partitions. (B) Confocal image stacks containing mostly the radial nerve from the images in Figure 12B. Arrows as described in (A); proximal limb to the left; scale bars = 250 µm. (C) Extensor muscles from Ark2C +/− Smad8 −/− adult mice. ECRL, extensor carpi radialis longus; ECRB, extensor carpi radialis brevis; EDC, extensor digitorum communis; EDQ, extensor digiti quinti; R, right limb; L, left limb; scale bars = 1 mm. No EDC or EDQ was recovered from this Ark2C +/− Smad8 −/− mouse. (TIF) [file pbio.1001538.s008.tif]

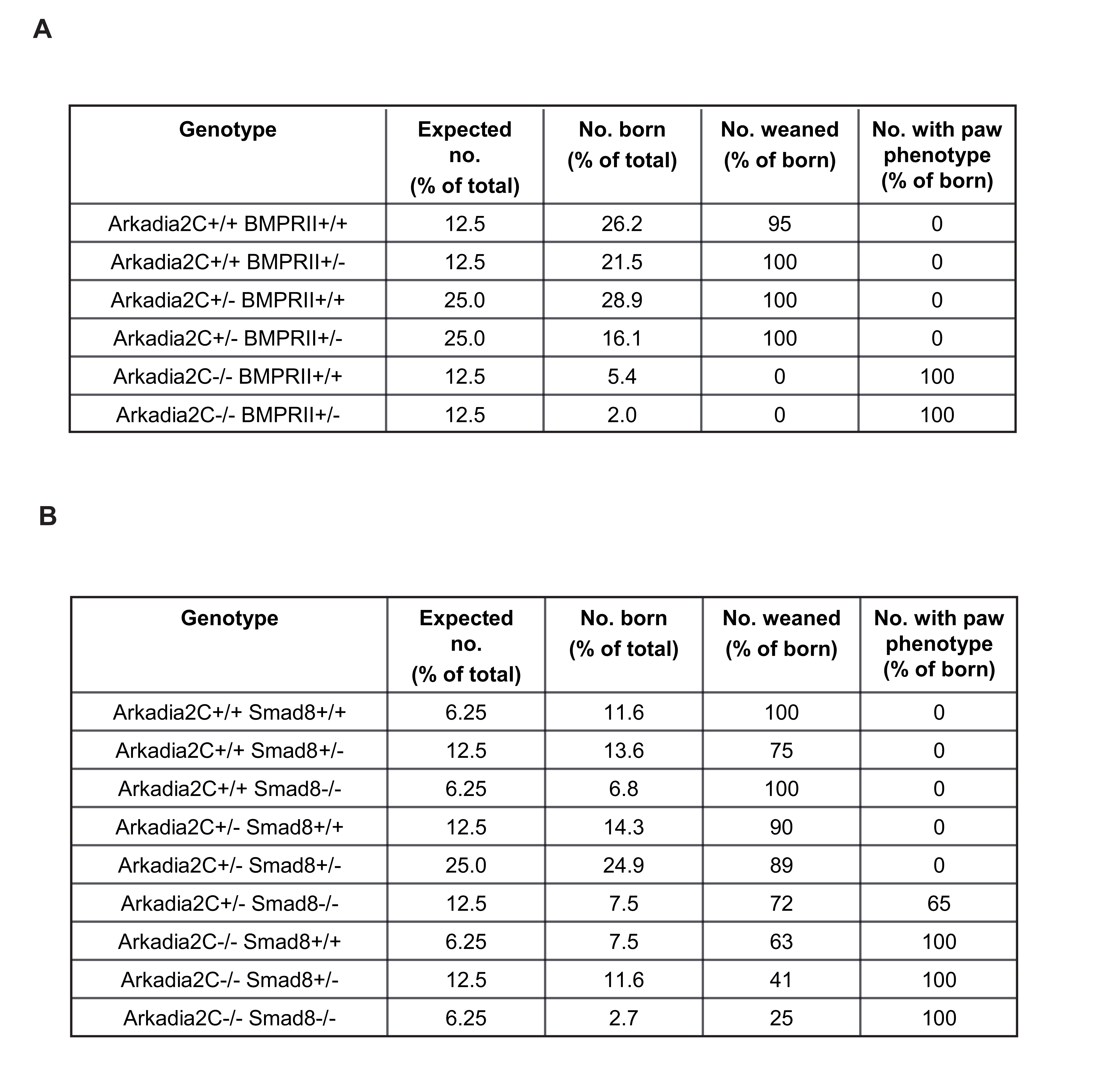

Supplement: Table S1 — Survival rates of offspring from genetic interactions with Ark2C. (A) Ark2C +/−:BmprII +/−×Ark2C +/− cross and (B) Ark2C+/−:Smad8+/−×Ark2C+/−:Smad8+/− cross. Tables show expected percentage of total births (according to Mendelian ratios) for each genotype, the actual percentage of total births observed (all animals alive less than 24 h after birth), and the percentage of those animals born that reach weaning age (21 d). p<0.001 for numbers born in Ark2C/BmprII cross including and excluding Ark2C −/− pups (χ2 test, 5 and 3 degrees of freedom, respectively), and p<0.01 for numbers weaned in Ark2C/Smad8 cross including and excluding Ark2C −/− pups (χ2 test, 8 and 5 degrees of freedom, respectively). (TIF) [file pbio.1001538.s009.tif]

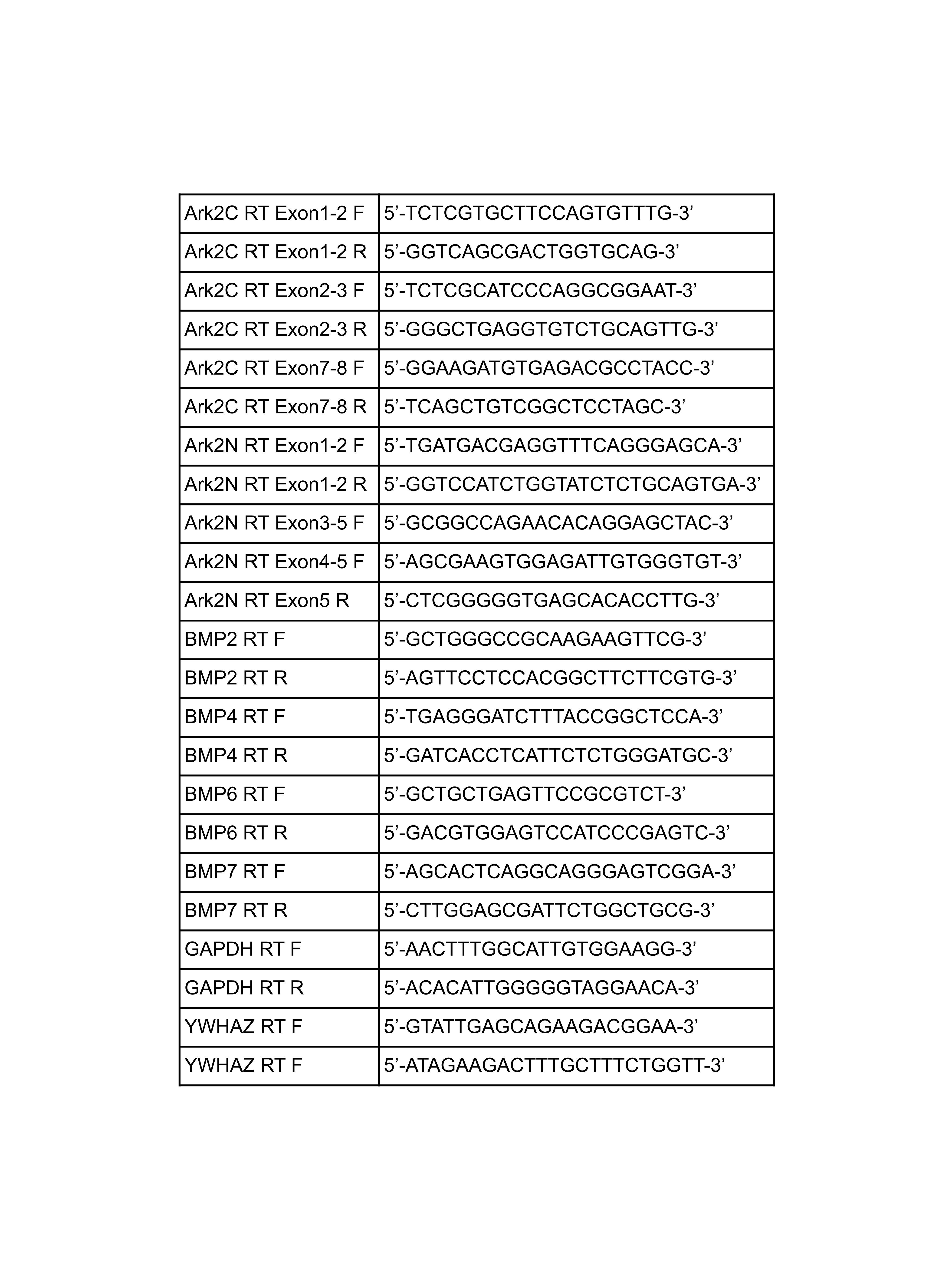

Supplement: Table S2 — Quantitative PCR primers. Sequences of primers used in all quantitative and semiquantitative RT-PCR. (TIF) [file pbio.1001538.s010.tif]
